# Co-inhibition of BET proteins and NF-κB as a potential therapy for colorectal cancer through synergistic inhibiting MYC and FOXM1 expressions

**DOI:** 10.1038/s41419-018-0354-y

**Published:** 2018-02-22

**Authors:** Tingyu Wu, Guanghui Wang, Wei Chen, Zhehui Zhu, Yun Liu, Zhenyu Huang, Yuji Huang, Peng Du, Yili Yang, Chen-Ying Liu, Long Cui

**Affiliations:** 1 Department of Colorectal Surgery, Xinhua Hospital, Shanghai Jiao Tong University School of Medicine, Shanghai, China; 2 Shanghai Colorectal Cancer Research Center, Shanghai, China; 3 Department of Gastroenterology, Hepatology and Nutrition, The University of Texas MD Anderson Cancer Center, Houston, TX USA; 4 Suzhou Institute of Systems Medicine, Center for Systems Medicine, Chinese Academy of Medical Sciences, Suzhou, China

## Abstract

The bromodomain and extra-terminal domain inhibitors (BETi) are promising epigenetic drugs for the treatment of various cancers through suppression of oncogenic transcription factors. However, only a subset of colorectal cancer (CRC) cells response to BETi. We investigate additional agents that could be combined with BETi to overcome this obstacle. JQ1-resistant CRC cells were used for screening of the effective combination therapies with JQ1. RNA-seq was performed to explore the mechanism of synergistic effect. The efficacy of combinational treatment was tested in the CRC cell line- and patient-derived xenograft (PDX) models. In BETi-sensitive CRC cells, JQ1 also impaired tumor angiogenesis through the c-myc/miR-17-92/CTGF+THBS1 axis. CTGF knockdown moderately counteracted anti-angiogenic effect of JQ1 and led to partially attenuated tumor regression. JQ1 decreased c-myc expression and NF-κB activity in BETi-sensitive CRC cells but not in resistant cells. Bortezomib synergistically sensitized BETi-resistant cells to the JQ1 treatment, and JQ1+Bortezomib induced G2/M arrest in CRC cells. Mechanistically, inhibition of NF-κB by Bortezomib or NF-κB inhibitor or IKK1/2 siRNA all rendered BETi-resistant cells more sensitive to BETi by synergistic repression of c-myc, which in turn induces GADD45s’ expression, and by synergistic repression of FOXM1 which in turn inhibit G2/M checkpoint genes’ expression. Activation of NF-κB by IκBα siRNA induced resistance to JQ1 in BETi-sensitive CRC cells. Last, JQ1+Bortezomib inhibited tumor growth and angiogenesis in CRC cell line xenograft model and four PDX models. Our results indicate that anti-angiogenic effect of JQ1 plays a vital role in therapeutic effect of JQ1 in CRC, and provide a rationale for combined inhibition of BET proteins and NF-κB as a potential therapy for CRC.

## Introduction

In CRC, dysregulation of the epigenome has been recognized as one of the major drivers of tumorigenesis and tumor progression^1^. One of the most promising epigenetic targets are the bromodomain and extra-terminal domain (BET) family proteins (BRD2, BRD3, BRD4, and BRDT). BET inhibitors (BETi), such as JQ1, can suppress transcription of a number of oncogenes, particularly that regulated by super-enhancers such as c-myc, FOS, and JUNB^2^. BETi was first found to have great efficacy in hematological malignancies by repressing c-myc expression^3,4^, and then showed promising responses in preclinical models of various cancers^5–8^. In colorectal cancer, JQ1 also induced c-myc downregulation and growth inhibition in a subset of CRC with high CCAT1 expression^9^. However, the responsiveness to BETi appeared to be very heterogeneous in CRC. The intrinsic JQ1-resistant mechanism and strategy to overcome drug resistance are still need to be explored.

In this study, we explored the therapeutic potential of BETi in CRC and investigated the underlying mechanisms conferring to BETi resistance. We revealed that blockade of the NF-κB pathway by Bortezomib, a 20S proteasome inhibitor and FDA-approved drug for multiple myeloma and mantle cell lymphoma^10^, could render CRC more sensitive to BETi, through synergistic repression of c-myc and FOXM1. Our results provide a rational basis for the combination therapy using inhibitors for BET proteins and NF-κB pathway in CRC.

## Results

### Bortezomib synergistically sensitizes BETi-resistant cells to JQ1 treatment

**Fig. 1:**
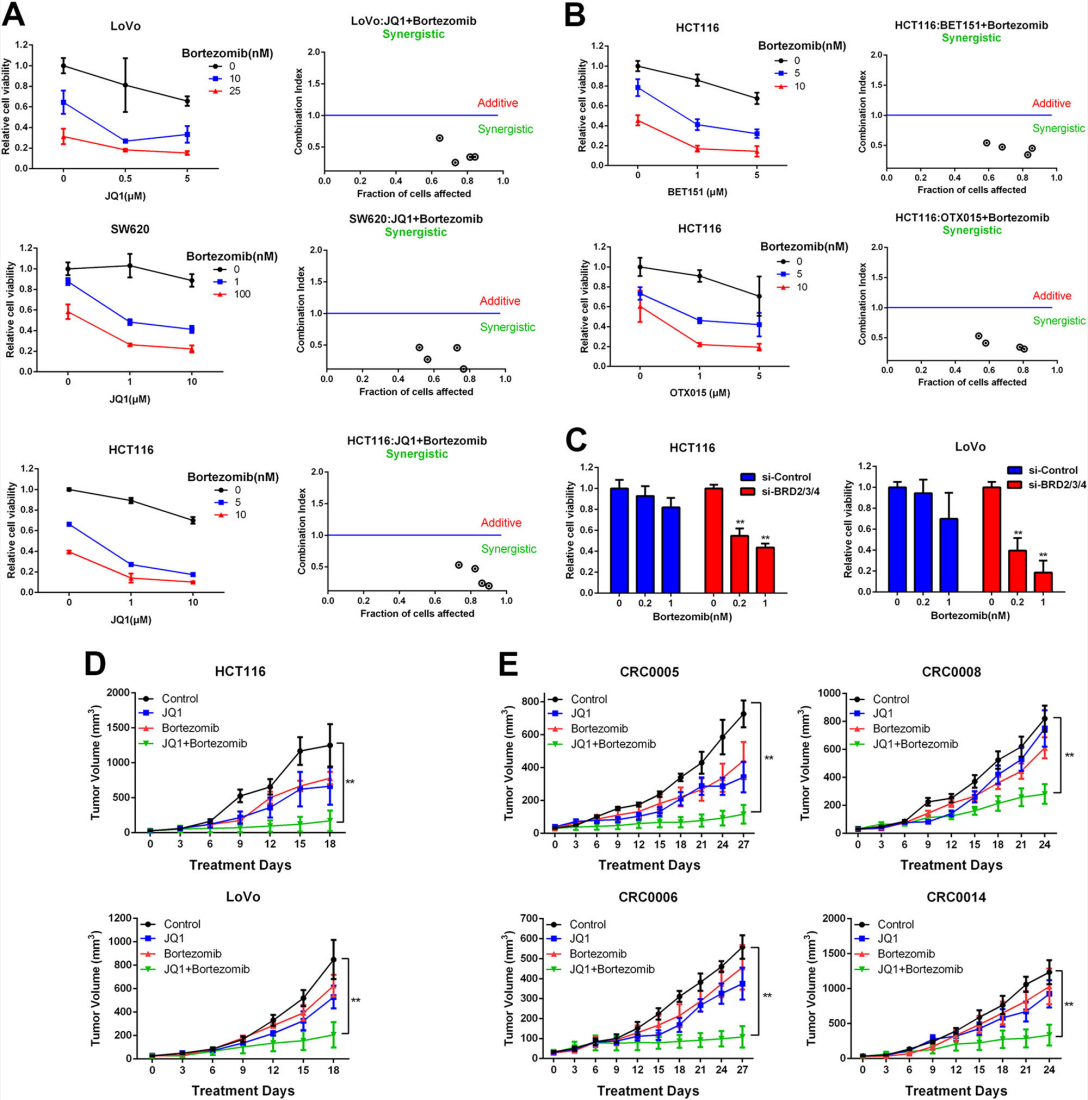
JQ1 and Bortezomib exhibit synergistic effect in BETi-resistant CRC cells. **a** BETi-resistant cells were co-treated with JQ1 and Bortezomib at the indicated concentrations for 72 h. Cell viability was measured by CCK8. The synergistic cytotoxicity was quantitatively analyzed by combination index (CI) using the Calcusyn software program. Each dot represented one combinational treatment group. CI > 1 indicates additive effect, and CI < 1 indicates synergistic effect. **b** HCT116 cells were treated with BET151 or OTX015 in the presence of bortezomib for 72 h. Cell viability and CI values were as indicated. **c** LoVo and HCT116 cells were transfected with BRD2/3/4 siRNA for 2 days, then cells were treated with bortezomib for another 72 h. Cell viability was measured by CCK8. **d** Growth curves of HCT116 and LoVo xenograft tumors treated with vehicle (control), JQ1, Bortezomib, or JQ1+Bortezomib for 18 days. **e** Growth curves of four CRC patients-derived xenograft tumors treated with vehicle (control), JQ1, Bortezomib, or JQ1+Bortezomib for 27 days

To explore the anti-tumor activity of BET inhibition in CRC cells, we treated a panel of 11 CRC cell lines with different BETi (Supplementary Fig. 1A–D). Consistent to a previous report^9^, we found that a subset of cell lines (LoVo, SW620, DLD1, and HCT116) was resistant to all the BETi. The minimal response to BET inhibitors in the resistant cells suggest intrinsic resistance to BET inhibitors in CRC, this led us to investigate additional agents that could be combined with JQ1 to overcome this obstacle. We selected seven drugs including conventional chemotherapeutic drugs and inhibitors that target epigenetic regulator, canonical cancer-related pathways (NF-κB, Hippo, MAPK, and PI3K), and established cell culture and CI (combination index) value assay^11^ to screen for the effective combination therapies in the BETi-resistant cells (Fig. 1a, b and Supplementary Fig. 2). Intriguingly, proteasome inhibitor Bortezomib (BOR) showed dramatically synergistic effect with JQ1, BET151, or OTX015 in the BETi-resistant cells (CI < 1). Consistently, BRD2/3/4 knockdown significantly enhanced the cytotoxic effect of Bortezomib (Fig. 1c).

Next, we examined the synergistic effect of JQ1 and Bortezomib in the xenograft model. The results showed that the combinational treatment significantly enhanced tumor growth regression compared with vehicle or individual drug treatment in both HCT116 and LoVo cells (Fig. 1d and Supplementary Fig. 3A, B). To further evaluate the translational therapeutic potential of JQ1 and Bortezomib co-treatment, we established four patient-derived primary human CRC xenografts (PDX model) in our study (Supplementary Fig. 3C). The clinical pathological features were shown in Supplementary Fig. 3E. Tumor growth of four PDXs with JQ1 and Bortezomib co-treatment and single-drug treatment were measured (Fig. 1e and Supplementary Fig. 3D, F). JQ1 treatment alone led to moderate tumor regression (53.1 and 33.6% reduction) in model CRC0005 and CRC0006, which were identified as partial responsive (+, PR) to JQ1 according to RICIST criterion^12^. Whereas Bortezomib co-treatment with JQ1 further inhibited tumor growth (82.7 and 80.9% reduction), which reached complete responsive (++, CR). For another two patients (CRC0008 and CRC0014) who were resistant to FOLFOX6 therapy, their xenografts were non-responsive (-) to JQ1 treatment (7.4 and 26.2% reduction). Intriguingly, Bortezomib still sensitized resistant xenografts to JQ1 treatment with partial (64.1% reduction, CRC0008) and complete response (73.4% reduction, CRC0014) respectively, suggesting that combinational treatment with JQ1 and Bortezomib exerts the promising anti-tumor activity in the JQ1- or FOLFOX-resistant patients.

### JQ1 and Bortezomib co-treatment induce growth inhibition, apoptosis, and G2/M arrest in BETi-resistant cells

**Fig. 2:**
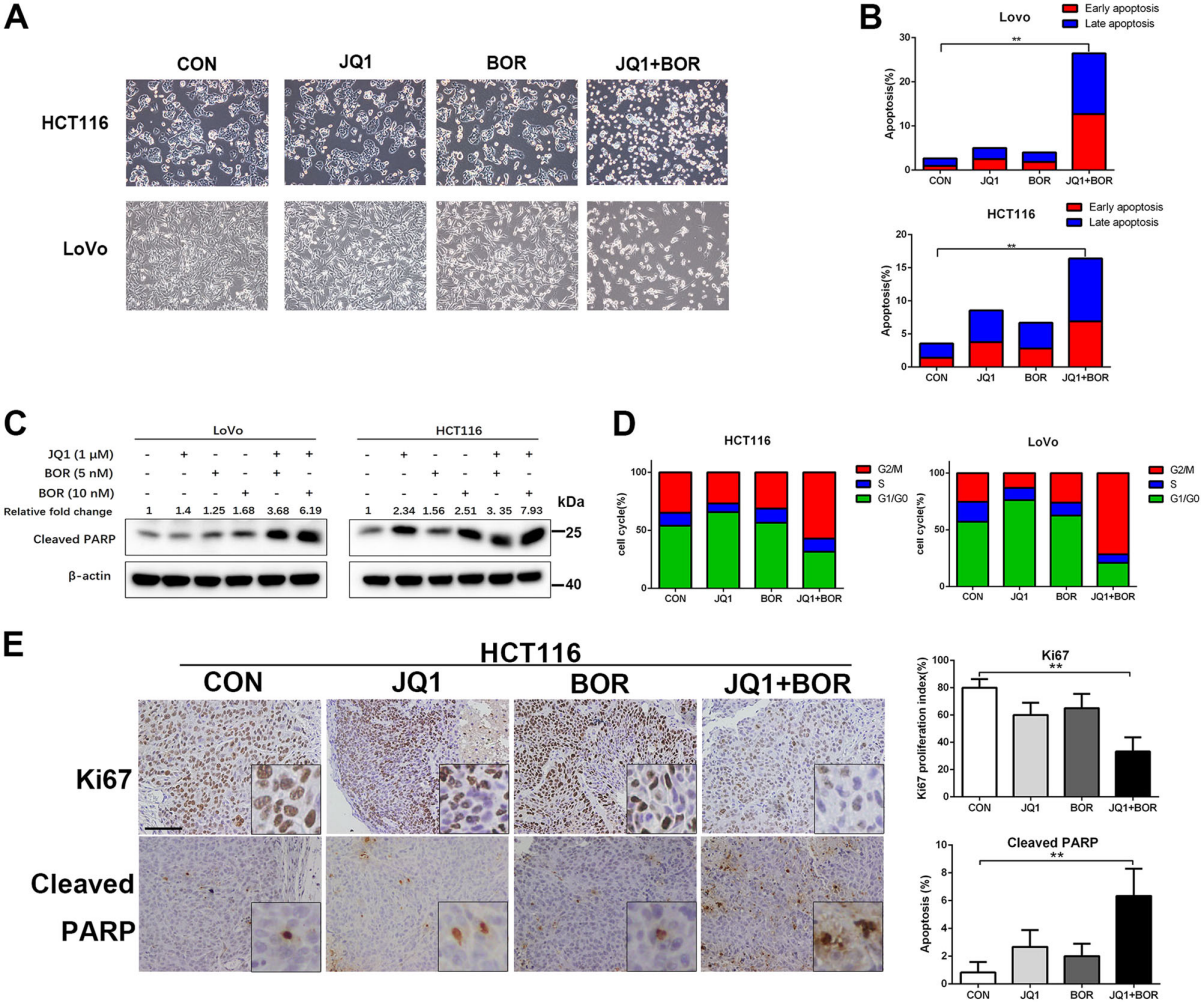
JQ1 and Bortezomib exhibit synergistic cytotoxicity in BETi-resistant cells. **a** Representative phase contrast microscope images of LoVo and HCT116 cells treated with JQ1 (1 μM), Bortezomib (5 nM), or JQ1+Bortezomib for 72 h. **b** LoVo and HCT116 cells were treated with JQ1 (1 μM), Bortezomib (5 nM), or JQ1+Bortezomib for 48 h. Cell apoptosis was analyzed by Annexin V/PI staining. **c** Western blot of cleaved PARP1 in the HCT116 and LoVo cells treated with indicated compounds. **d** LoVo and HCT116 cells were treated with JQ1 (1 μM), Bortezomib (5 nM), or JQ1+Bortezomib for 48 h. Cell cycles were analyzed by PI staining. **e** Immunohistochemical staining of Ki67, cleaved PARP in xenograft tumor sections of HCT116 cells. Bar, 100 μm

Combined treatment with JQ1 and Bortezomib induced dramatically cell shrinkage, increased apoptosis, and elevated cleaved PARP expression in the LoVo and HCT116 BETi-resistant cells (Fig. 2a–c). Interestingly, combination of JQ1 and Bortezomib markedly induced G2/M arrest in BETi-resistant cells (Fig. 2d). In vivo, the combination treatment also resulted in significantly reduced level of cell growth (Ki67) and increased apoptosis (cleaved PARP) in the HCT116 xenograft and PDX model (Fig. 2e and Supplementary Fig. 8). Collectively, our results indicate that proteasome inhibitor Bortezomib and BET inhibitor synergized to suppress tumor growth in the BETi-resistant cells.

### JQ1 and Bortezomib treatment synergistically induces G2/M arrest through inhibiting FOXM1 expression

**Fig. 3:**
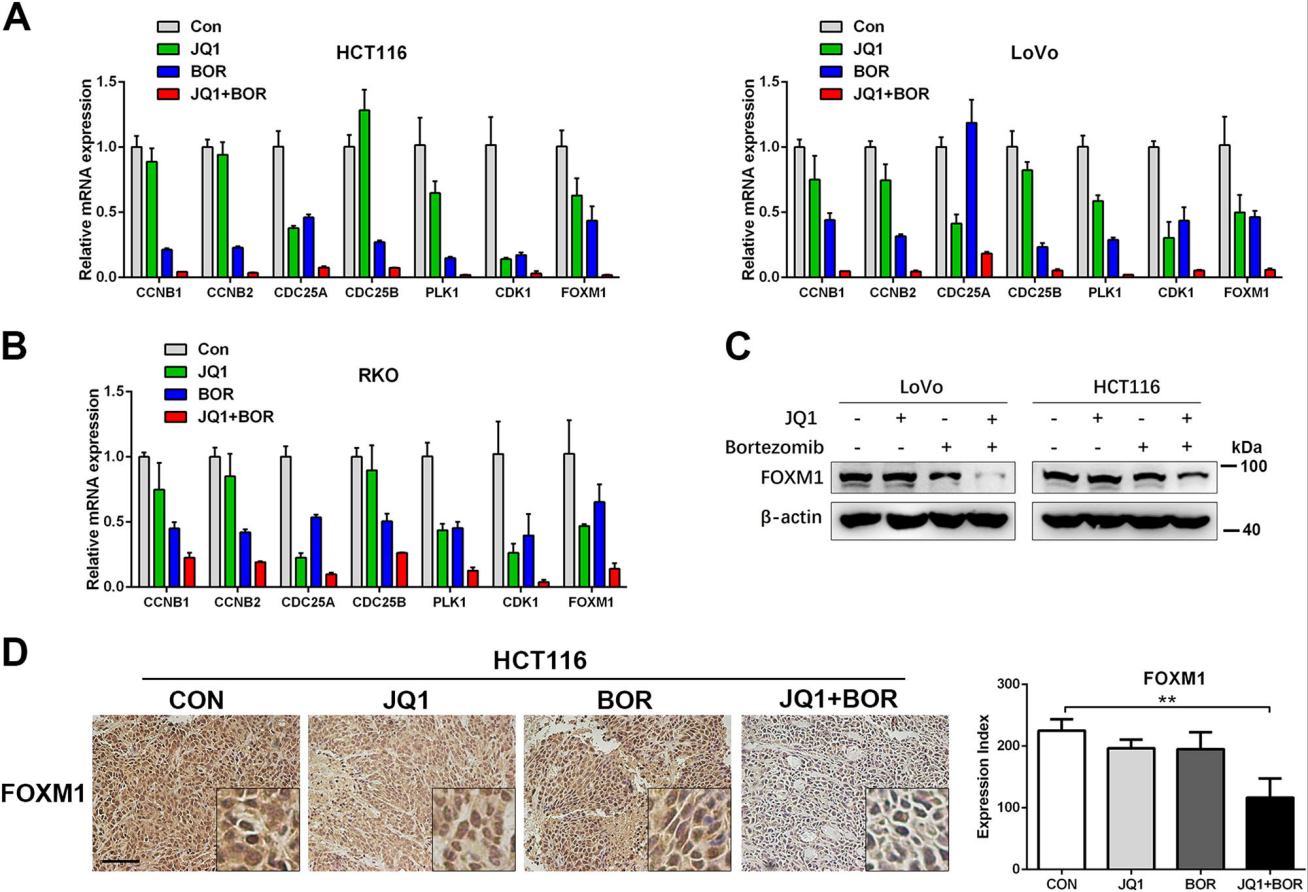
JQ1 and Bortezomib synergistically induce G2/M arrest through inhibiting FOXM1 expression. **a**, **b** qPCR analysis of G2/M checkpoint genes’ expression in HCT116, LoVo, and RKO cells after the treatment of indicated chemicals. **c** Western blot analysis of FOXM1 protein expression in HCT116 and LoVo cells after the treatment of indicated chemicals. **d** Immunohistochemical staining of FOXM1 in xenograft tumor sections of HCT116 cells. Bar, 100 μm

JQ1+Bortezomib induced G2/M arrest in three BETi-resistant cells (Fig. 2d and Supplementary Fig. 4C). Interestingly, though JQ1 single agent induced G1 arrest in the RKO cells, co-treatment of JQ1 and Bortezomib induced G2/M arrest in RKO cells (Supplementary Fig. 4C). To investigate the molecular mechanism underlying the synergistic effect of JQ1 and Bortezomib, we analyzed gene expression profiles of HCT116 cells treated with DMSO, JQ1, Bortezomib, or JQ1+Bortezomib. A set of 102 significantly differentially expressed genes in the JQ1+Bortezomib group that are essential in tumor proliferation and survival were shown in Supplementary Fig. 4C. GO analysis indicated that co-treatment with JQ1 and Bortezomib significantly inhibited cell cycle-related gene expression (Supplementary Fig. 4B). Specifically, RNA-seq analysis showed that the expression of several cell cycle genes related with G2/M arrest (FOXM1, PLK1, CDC25B, CDK1, CCNB1, and CCNB2) was significantly suppressed after JQ1+Bortezomib treatment (Supplementary Fig. 4A), which is confirmed by qPCR in both HCT116 and LoVo cells (Fig. 3a). In HCT116 and LoVo cells, JQ1 treatment did not affect G2/M arrest genes’ mRNA expression and Bortezomib treatment mild inhibit mRNA expressions of these G2/M arrest genes. However, G2/M arrest genes’ expression were also totally abolished after JQ1+Bortezomib co-treatment. Similar results were observed in the RKO cells (Fig. 3b). The western blot and IHC analysis also showed that JQ1 and Bortezomib co-treatment synergistically inhibited FOXM1 protein expression in vitro and in vivo, while JQ1 or Bortezomib single drug did not affect FOXM1 protein expression (Fig. 3c, d and Supplementary Fig. 8).

FOXM1 has been known for the crucial role for the G2/M transition whose target genes include cyclin B, Cdc25B, and Polo-like kinase^13^. GESE analysis showed that Bortezomib single reagent or co-treatment with JQ1 all suppressed gene expression of FOXM1 pathway (Supplementary Fig. 4G). To test whether FOXM1 is the key node for the G2/M arrest induced by JQ+Bortezomib co-treatment, two independent FOXM1 siRNA were used to knockdown FOXM1 in HCT116 cells (Supplementary Fig. 4D). Knockdown of FOXM1 resulted in downregulation of other G2/M transition genes (Supplementary Fig. 4E) and G2/M arrest in HCT116 cells (Supplementary Fig. 4F). These data indicate that decreased expression of FOXM1 is the key event after JQ1+Bortezomib co-treatment.

### GADD45 plays a critical role in synergistic cytotoxic effect

**Fig. 4:**
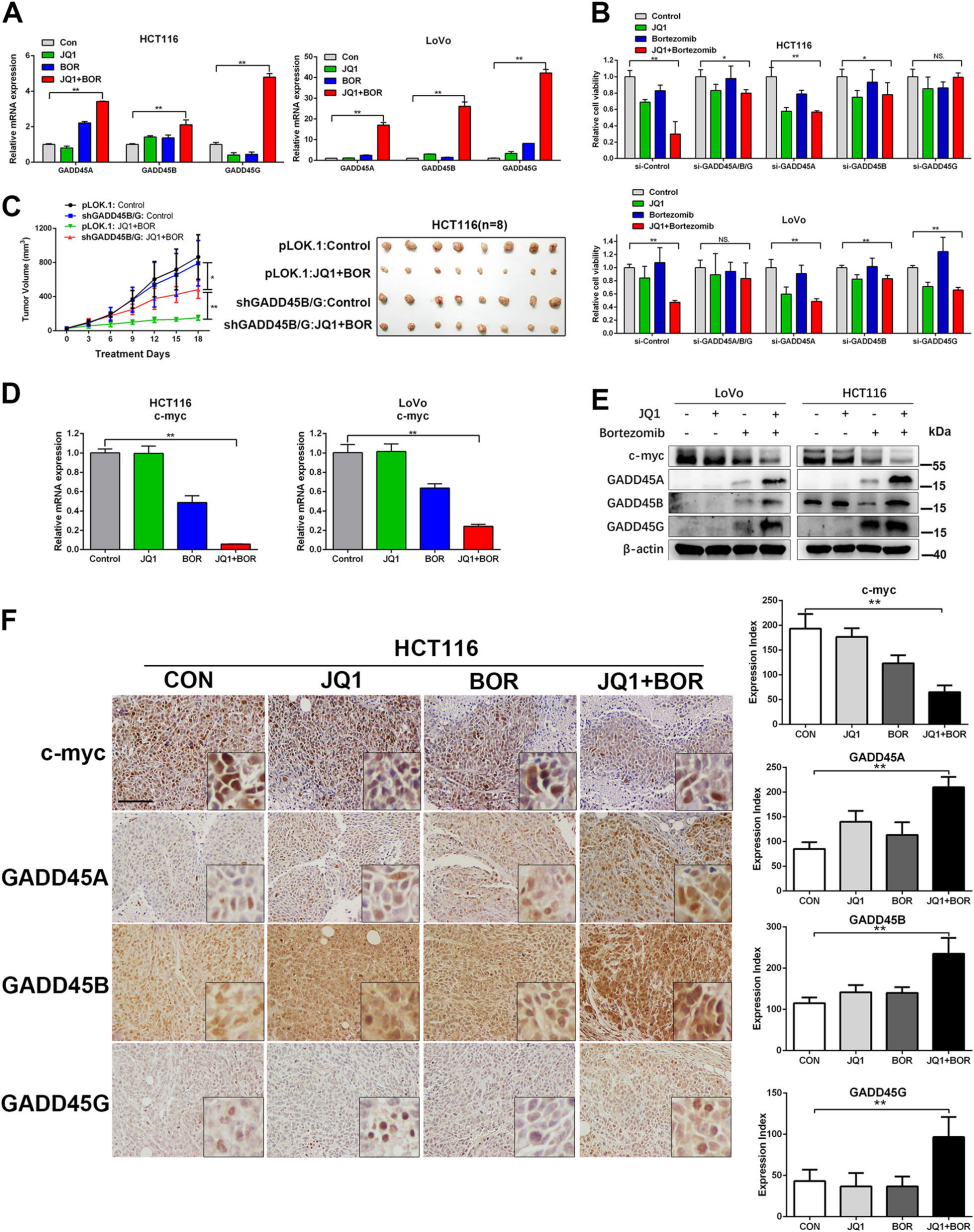
JQ1 and Bortezomib induce synergistic effect through upregulation of GADD45. **a** mRNA alteration of GADD45 in HCT116 and LoVo cells treated with JQ1 (1 μM), Bortezomib (5 nM), or JQ1+Bortezomib for 24 h. **b** LoVo and HCT116 cells were transfected with GADD45A/B/G siRNA for 2 days, then cells were treated with indicated compounds for another 72 h. Cell viability was measured by CCK8. **c** Xenograft growth curves of shGADD45B/G HCT116 stable cells treated with vehicle (control), JQ1, Bortezomib, or JQ1+Bortezomib for 18 days. **d** mRNA alteration of c-myc in HCT116 and LoVo treated with JQ1 (1 μM), Bortezomib (5 nM), or JQ1+Bortezomib for 24 h. **e** Western blot analysis of HCT116 and LoVo cells treated with JQ1 (1 μM), Bortezomib (5 nM), or JQ1+Bortezomib for 24 h. **f** Immunohistochemical staining of c-myc, GADD45A/B/G in xenograft tumor sections of HCT116 cells. Bar, 100 μm

Intriguingly, we noticed that three GADD45 family genes (GADD45A/B/G) were all synergistically significantly upregulated by treatment of JQ1 and Bortezomib (Supplementary Fig. 4A). GADD45 proteins are well known as tumor suppressors. The anti-cancer activity of chemotherapeutic agents relies on GADD45 upregulation for induction of G2/M cell cycle arrest and apoptosis in tumor cells^14^, promoting us to explore whether induction of GADD45 proteins play a critical role in the synergistic effect of combinational treatment. We first confirmed GADD45 expressions were dramatically increased in the JQ1+Bortezomib group but not in the single drug treatment group by qPCR (Fig. 4a). To further determine the pivotal role of GADD45 in the synergistic effect, we used siRNA to knockdown GADD45 which was confirmed by qPCR (Supplementary Fig. 5A). GADD45 knockdown, especially for GADD45B and GADD45G, markedly abolished the synergistic cytotoxic effect of JQ1 and Bortezomib in both LoVo and HCT116 (Fig. 4b). To test the functions of GADD45 in vivo, HCT116 cells expressing both GADD45B and GADD45G shRNA were generated (Supplementary Fig. 5B). Knockdown of GADD45B/G largely abolished synergistic anti-tumor effect of JQ1 and Bortezomib co-treatment in vivo (Fig. 4c).

c-myc has been shown to repress expression of the GADD45 family members^15–17^, thus we explored whether GADD45 proteins are also involved in the cytotoxic effect of JQ1 in the BETi-sensitive cells. We found that knockdown of c-myc led to significant increase of GADD45A/B/G in the RKO cells (Supplementary Fig. 5C), suggesting that expression of GADD45 family was regulated by c-myc in CRC. JQ1 treatment induced potent repression of c-myc and increased expression of GADD45A/B/G in BETi-sensitive cells (RKO, HT29, SW480, SW48, and LS174T) but not in the BETi-resistant cells (HCT116, LoVo, and SW620) (Supplementary Fig. 5D–F). Moreover, in BETi-sensitive RKO cells, cell death induced by JQ1 were largely attenuated by GADD45A/B/G knockdown (Supplementary Fig. 5G), further indicating that cytotoxic effect of JQ1 was mediated by GADD45. These findings prompted us to investigate the effect of JQ1 and Bortezomib co-treatment on c-myc expression in the BETi-resistant cells. Bortezomib single treatment mildly repressed c-myc expression in the HCT116 and LoVo cells at mRNA level and protein level (Fig. 4d, e). Strikingly, JQ1 and Bortezomib treatment synergistically inhibited c-myc expression, which in turn induced marked GADD45 expression in both HCT116 and LoVo cells (Fig. 4e). JQ1 and Bortezomib co-treatment also significantly reduced level of c-myc and increased expression of GADD45A/B/G, compared with vehicle or either drug alone in the HCT116 xenografts (Fig. 4f). Our results indicate that synergistic effect of JQ1 and Bortezomib rely on the induction of GADD45 proteins through repressing c-myc expression in the BETi-resistant CRC cells.

### Bortezomib sensitizes resistant cells to JQ1 treatment through blocking NF-κB pathway

**Fig. 5:**
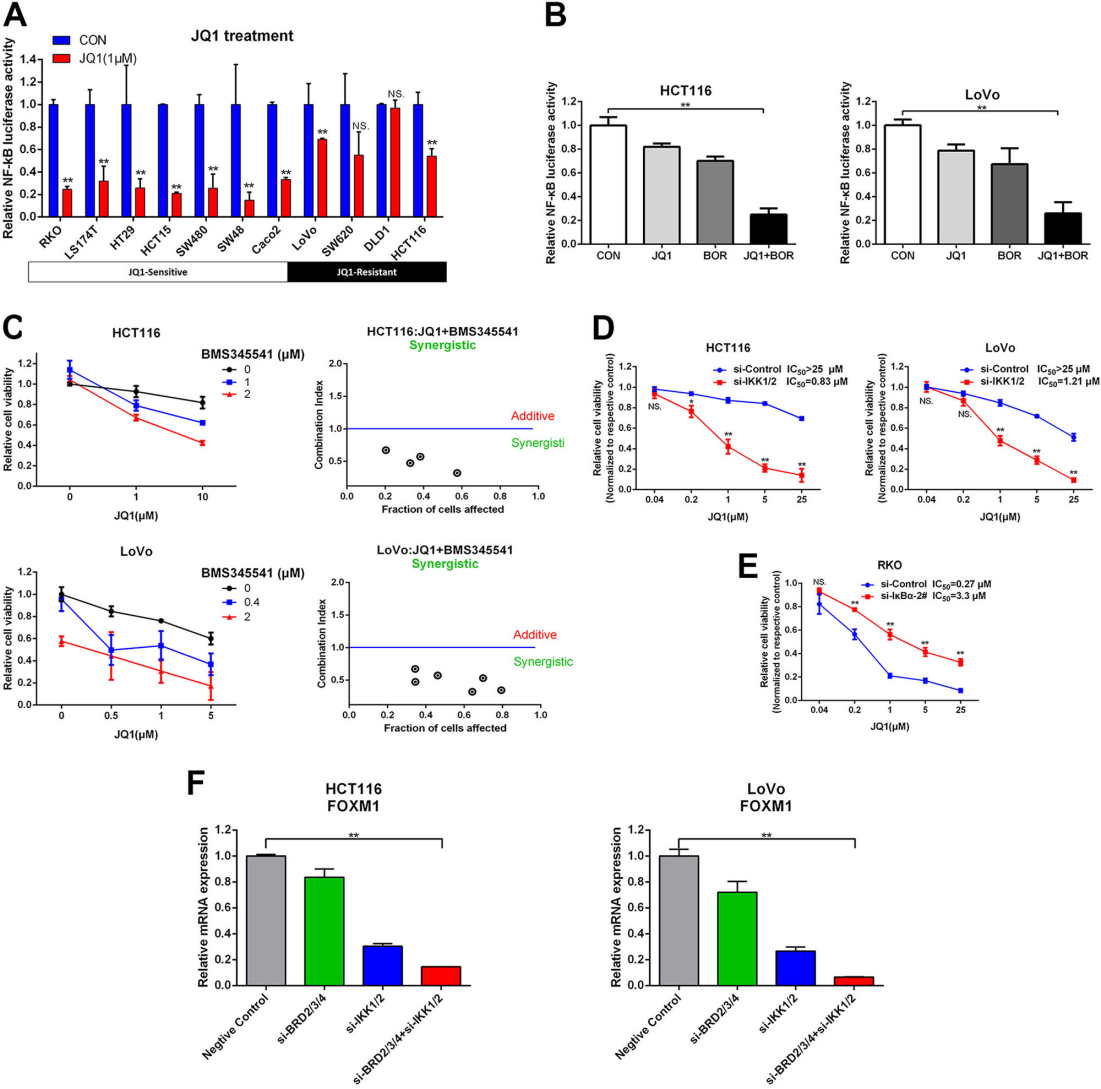
Blocking of NF-κB pathway sensitizes BETi-resistant CRC cells to JQ1 treatment. **a** CRC cells were treated with JQ1 (1 μM) for 24 h and subjected to NF-κB reporter assay. **b** HCT116 and LoVo cells were treated with JQ1 (1 μM), Bortezomib (5 nM), or JQ1+Bortezomib for 24 h and subjected to NF-κB reporter assay. **c** HCT116 and LoVo were treated concurrently with JQ1 and BMS345541 at the indicated concentrations for 72 h. Cell viability was measured by CCK8. The synergistic cytotoxicity was quantitatively analyzed by CI. **d** HCT116 and LoVo cells were transfected with IKK1/2 siRNA for 2 days, then cells were treated with JQ1 (1 μM) for another 72 h. Cell viability was measured by CCK8. **e** RKO cells were transfected with IκBα siRNA for 2 days, then cells were treated with JQ1 (1 μM) for another 72 h. Cell viability was measured by CCK8. **f** mRNA alteration of FOXM1 in HCT116 and LoVo cells transfected with BRD2/3/4, IKK1/2, or BRD2/3/4+IKK1/2 siRNA for 72 h

Bortezomib is a selective proteasome inhibitor affecting multiple signaling pathways including NF-κB pathway^18^. It is well known that c-myc promoter is transactivated by NF-κB^19^, prompting us to explore whether NF-κB is the primary target of Bortezomib synergizing with JQ1 in CRC. First, we confirmed the inhibition of Bortezomib on NF-kB activity by using NF-κB-dependent luciferase gene reporter. Bortezomib led to significant inhibition of NF-κB luciferase activity in both BETi-sensitive (RKO) and -resistant cells (SW620, LoVo, and HCT116) in a dose-dependent manner (Supplementary Fig. 6A). BRD4 can promote the transcriptional activation of NF-κB by binding to acetylated RelA^20,21^. In CRC, NF-κB activity was dramatically repressed by JQ1 treatment alone in BETi-sensitive cells (Fig. 5a). However, JQ1 only led to minimal inhibition of NF-κB activity in JQ1-resistant cells (Fig. 5a). Dramatic repression of NF-κB activity was observed in both LoVo and HCT116 with JQ1+Bortezomib compared to single drug treatment (Fig. 5b). To further confirm NF-κB inhibition by Bortezomib is involved in synergistic effect, another NF-κB selective inhibitor BMS345541 was used. Similar synergistic effect of JQ1 and BMS345541 was observed in LoVo and HCT116 cells (Fig. 5c). JQ1 and BMS345541 co-treatment also induced decreased expression of c-myc and upregulation of GADD45A/B/G in HCT116 cells (Supplementary Fig. 6B). Furthermore, we used IKK1/2 siRNA (Supplementary Fig. 6C) to specific inhibit NF-κB activity (Supplementary Fig. 6E). We found that knockdown of IKK1/2 downregulated expression of c-myc (Supplementary Fig. 6F) and sensitized the HCT116 and LoVo cells to the treatment of JQ1 (Fig. 5d). We also used IκBα siRNA (Supplementary Fig. 6D) to activate NF-κB pathway (Supplementary Fig. 6G). Consistently, knockdown of IκBα in the BETi-sensitive RKO cells rendered cells to resist JQ1-induced cytotoxicity (Fig. 5e).

**Fig. 6:**
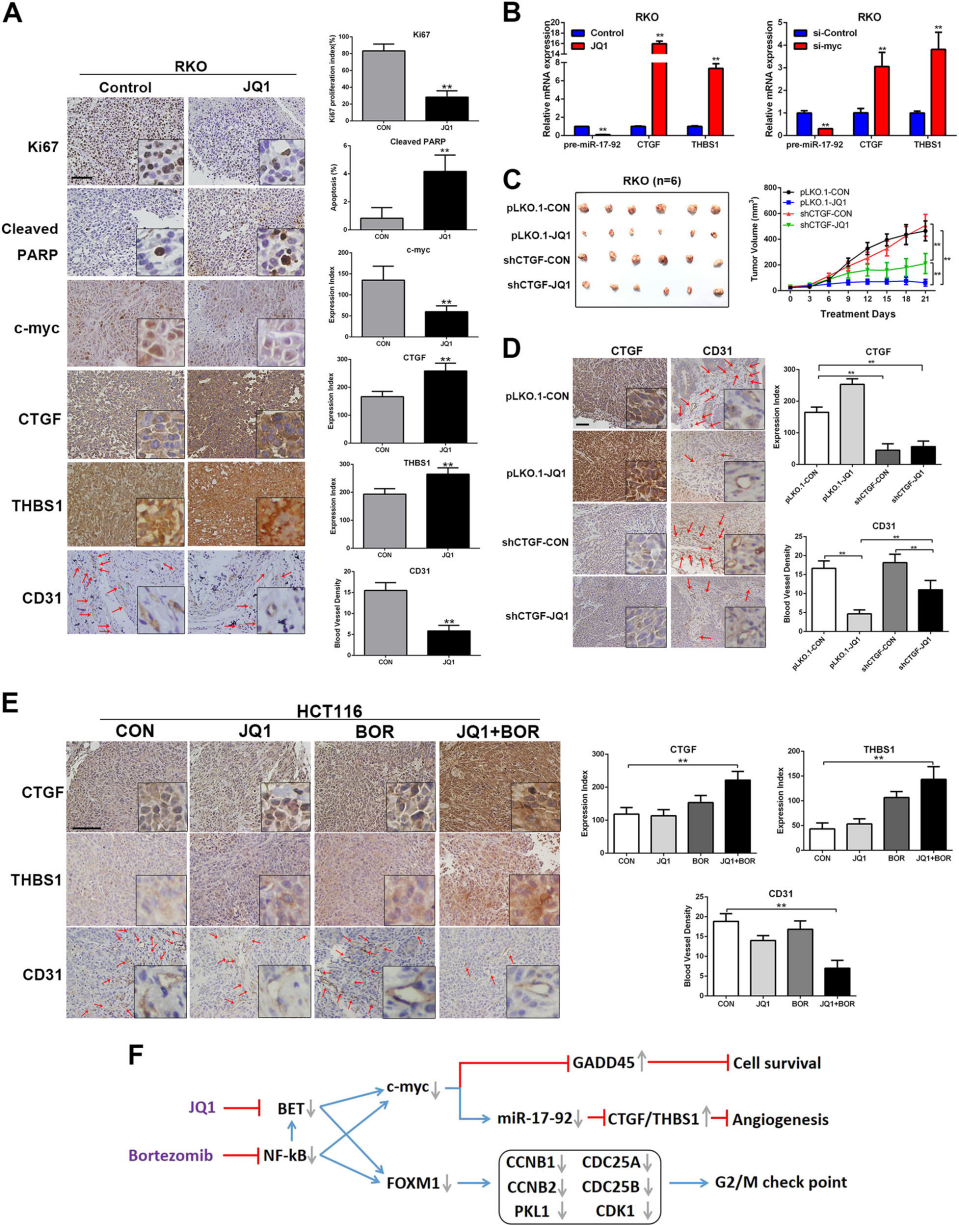
JQ1 and Bortezomib synergistically inhibits angiogenesis in BETi-resistant CRC cells. **a** Immunohistochemical staining of Ki67, cleaved PARP, c-myc, CTGF, THBS1, and CD31 in the xenograft tumors of RKO cells. **b** Relative mRNA expression of pre-miR-17-92, CTGF, and THBS1 in RKO cells was detected by qPCR after JQ1 treatment or c-myc siRNA transfection. **c** Xenograft growth curves of pLKO.1 control and shCTGF RKO stable cells treated with JQ1 (50 mg/kg, daily) or vehicle control for 21 days. Bar, 100 μm. **d** Immunohistochemical staining of CTGF, and CD31 in the xenograft tumors of RKO cells. **e** Immunohistochemical staining of CTGF, THBS1, and CD31 in the xenograft tumors of HCT116 cells. Bar, 100 μm. **f** A proposed model for synergistic effect of JQ1 and Bortezomib co-treatment in CRC

Silence of NF-κB reduced the expression of FOXM1 in breast cancer cells^22^. Recently, FOXM1 has been reported as a functional target of BETi in ovarian cancer^23^. These findings prompted us to explore whether inhibition of BRD and NF-κB synergistically suppress FOXM1 expression in the CRC cells. First, we found that JQ1 and BMS345541 co-treatment also induced G2/M arrest in the HCT116 cells (Supplementary Fig. 6H). Knockdown of BRD2/3/4 only weakly decreased FOXM1 mRNA level, and inhibition of NF-κB activity by knockdown of IKK1/2 moderately inhibited FOXM1 mRNA expression (Fig. 5f). Simultaneous knockdown of BRD2/3/4 and IKK1/2 largely suppressed the FOXM1 mRNA expression (Fig. 5f). Above all, our results demonstrate that inhibition of NF-κB can sensitize BETi-resistant cells to BET inhibition by synergistic inhibition the expression of c-myc and FOXM1 (Fig. 6f).

### JQ1 and Bortezomib treatment synergistically inhibits angiogenesis in resistant cells

Subcutaneous xenograft models of JQ1-sensitive cells (RKO) treated with JQ1 were also assessed. As expected, JQ1 significantly reduced RKO tumor growth (85.26% tumor reduction, *p* < 0.01) in vivo (Supplementary Fig. 7A), suggesting efficient anti-tumor activity of JQ1 as monotherapy in JQ1-sensitive CRC. The tumor regression was associated with decreased proliferation (Ki67), increased apoptosis (cleaved PARP), and decreased expression of c-myc (Fig. 6a) in the JQ1 treatment group. Interestingly, as shown by CD31 staining, JQ1 treatment inhibited angiogenesis in the RKO xenograft tumors (Fig. 6a).

We also performed RNA-seq analysis to further assess the impact of JQ1 on gene expression profiles of RKO cells. Gene expression profiles identified that expression of CTGF and THBS1, two well-known anti-angiogenic genes inhibited by c-myc indirectly through promoting miR-17-92 expression in CRC^24^, were both upregulated upon JQ1 treatment in vitro (Supplementary Fig. 7B and Fig. 6b) and in vivo (Fig. 6a). qPCR analysis further confirmed that JQ1 resulted in reduced expression of pre-miR-17-92 and increased expression of CTGF and THBS1 in JQ1-sensitive cells (RKO, SW48, and LS174T) (Fig. 6b and Supplementary Fig. 7C) but not in the JQ1-resistent LoVo and HCT116 cells (Supplementary Fig. 7E). BRD2/3/4 or c-myc knockdown significantly reduced levels of pre-miR-17-92 and increased levels of CTGF and THBS1 in RKO, demonstrating the c-myc/miR-17-92/CTGF/THBS1 axis after JQ1 treatment (Fig. 6b and Supplementary Fig. 7C). To further explore whether anti-angiogenic effect also accounts for the anti-tumor activity of JQ1, shRNA was utilized to knockdown CTGF and THBS1 in RKO cells. However, as knockdown of THBS1 resulted in significant cell death, RKO cells only expressing shCTGF or empty vector were used in further study (Supplementary Fig. 7D). The xenograft assay showed that knockdown of CTGF moderately counteracted anti-angiogenic effect of JQ1 (Fig. 6d), and led to partially attenuated tumor regression (Fig. 6c). Our results indicate that anti-angiogenic effect of JQ1 plays a vital role in therapeutic effect of JQ1 in CRC.

Interestingly, gene expression profile analysis identified both CTGF and THBS1 were upregulated in the JQ1+Bortezomib group (Supplementary Fig. 4A). We confirmed the increased expression of CTGF and THBS1 in the JQ1+Bortezomib group in LoVo and HCT116 cells by qPCR (Supplementary Fig. 7E). JQ1 treatment did not affect CTGF and THBS1 expression in LoVo and HCT116 cells, while Bortezomib only mildly increase the expression of CTGF and THBS1 compared to the JQ1+Bortezomib group (Supplementary Fig. 7E). Consistently, JQ1+Bortezomib treatment dramatically repressed pre-miR-17-92 expression (Supplementary Fig. 7E). In vivo, blood vessel densities (BVD) decreased, and expression of both CTGF and THBS1 were increased by JQ1 and Bortezomib co-treatment (Fig. 6e), suggesting that combinational treatment also exerts anti-angiogenic effect in BETi-resistant cells.

Then, tumor sections of PDX models were also examined by IHC (Supplementary Fig. 8). Co-treatment led to a significant decrease in cell proliferation (Ki67), FOXM1, and c-myc expression, as well as increase in apoptosis and GADD45A/B/G expression (Supplementary Fig. 8). JQ1 treatment alone caused moderate deduction of BVD in CRC0005, whereas BVD was significantly decreased after combination treatment in PDX models (Supplementary Fig. 8). Taken together, these findings provide a primary data for the potential clinical translation of JQ1 and Bortezomib co-treatment regardless of JQ1 or FOLFOX responsiveness in colorectal cancer.

## Discussion

Transcription factor c-myc is a well-known oncogene activating or repressing transcription of a large number of genes involved cell proliferation, apoptosis, metabolism, and angiogenesis^25^. Overexpression of c-myc is observed in up to 70% of colorectal cancers^26,27^ and c-myc-dependent transcription program is ubiquitously activated in colorectal cancer^28^. As an important downstream target gene of β-catenin, targeted inactivation of c-myc impairs tumorigenesis induced by APC defect in the mouse CRC model^29^, indicating that c-myc is a promising drug target for colorectal cancer. Inhibition of c-myc expression is a plausible strategy to target c-myc indirectly. BET inhibitors have shown promising efficacy in hematopoietic and some solid tumors by downregulation c-myc expression^3^. Our results and a recent paper^30^ both show that the sensitivity of CRC cells to BETi is correlated with inhibition rate of c-myc expression, indicating that c-myc downregulation is the primary mechanism to inhibit tumor growth by BETi in CRC.

The minimal inhibition of c-myc expression by BETi in BETi-resistant cells indicates an intrinsic resistance to BETi, probably due to active c-myc expression by other transcription factors. Besides β-catenin, c-myc promoter is also transactivated by NF-κB^19^. BET inhibitor leads to preferential loss of BRD4 at super-enchancers^31^. The inhibition of c-myc expression is akin to the BRD4 depletion at the enchancers that drive c-myc expression^31^. Enhancer promoter interaction plays a vital role in activation of gene transcription^32^. BRD4 has been reported to bind with acetylated RelA to activate NF-κB transcriptional program^20,21^. It can be speculated that the interaction between BRD4 and NF-κB mediates the enhancer–promoter interaction of c-myc gene. Interestingly, our results show that JQ1 can more dramatically inhibit NF-κB activity in BETi-sensitive CRC cells than in resistant cells. This could account for the minimal inhibition of c-myc expression by BETi in BETi-resistant CRC cells. Thus, simultaneous inhibition of c-myc enhancer and promoter activity could account for synergistic effect of JQ1 and Bortezomib, and consequently result in full inhibition of c-myc gene transcription.

To explore the molecular basis of the synergistic effect of JQ1 and Bortezomib, we first identified that induction of GADD45 proteins play a vital role in the cell death induced by the combinational treatment. Knockdown of *GADD45* genes attenuates growth inhibition induced by combinational treatment. The *GADD45* gene family encodes three related GADD45 proteins, GADD45α, β, and γ, which are tumor suppressors implicating in regulation of many cellular functions including DNA repair, cell cycle control, apoptosis, senescence, and genotoxic stress^33^. c-myc can repress the expression of GADD45 through direct binding to GADD45 promoter^15–17^, and NF-κB can inhibit GADD45 expressions through activating c-myc expression^34^. Thus, increased expression of GADD45 with JQ1+Bortezomib treatment could be due to repression of c-myc expression, which is consistent to the observation in the JQ1 treatment or knockdown of c-myc in the BETi-sensitive cells all lead to increased expression of GADD45 proteins. We propose that induction of GADD45 proteins after c-myc inhibition are the key mediators of cell death upon JQ1 or JQ1+Bortezomib treatment.

BET inhibitor JQ1 inhibits tumor growth by inducing cell cycle arrest and apoptosis in cancer cells. Recently, mounting studies show that c-myc also affects tumor microenvironment and suppresses tumor angiogenesis^24,35^. We also found that, in colorectal cancer, JQ1 or JQ1+Bortezomib combinational treatment all inhibit angiogenesis. Inhibition of angiogenesis is also due to inhibition of c-myc expression, which consequently leads to reduced expression of miR-17-92 cluster and enhanced expression of two anti-angiogenic factors thrombospondin-1 (Tsp1 and THBS1) and connective tissue growth factor (CTGF)^24^. Since CTGF can be measured through blood biopsy, we suggest that serum CTGF may be used as biomarkers for how tumors respond to the JQ1 or JQ1+Bortezomib combinational treatment.

Bortezomib is a selective proteasome inhibitor which has been approved by FDA for the front-line treatment of multiple myeloma^18^. However, the efficacy of Bortezomib as a single agent in the solid tumors is limited^36^. In a clinical trial for metastatic colorectal cancer, Bortezomib failed to show significant antitumor activity^37^. This indicates that Bortezomib is ineffective in colorectal cancer as a single agent. Our finding that synergistic antitumor activity by BETi and Bortezomib in CRC provides the potential for the clinical translation of Bortezomib using in colorectal cancer.

The effect of proteasome inhibition is multifaceted and activates or represses multiple downstream signaling pathways including the NF-κB pathway^18^. IKK1/2 inhibitor and knockdown of IKK1/2 also sensitize CRC cells to JQ1 treatment and repress c-myc and FOXM1 expression, indicating that NF-κB is the primary target for Bortezomib to synergize with JQ1 to inhibit tumor growth. Activation of NF-κB in the BETi-sensitive cells attenuates the effect of BET inhibition, which further demonstrates activation of NF-κB could be the intrinsic mechanism for the resistance to the BETi in CRC. Biopsy of tumor tissues for analysis of active NF-κB could be a biomarker for the sensitivity for the BET inhibitors as a single agent in CRC.

Though we propose that inhibition of c-myc expression is important for the synergistic antitumor activity of JQ1 and Bortezomib, there are still other mechanisms of action. It is worth noting that JQ1+Bortezomib induces G2/M cell cycle arrest but not G1 arrest induced by JQ1 single agent in CRC cells. This indicates that inhibition of c-myc expression and GADD45 induction is not accounted for the G2/M arrest induced by combinational treatment, though c-myc^38^ and GADD45^39^ have been reported to be involved in the DNA damage-induced G2/M arrest. FOXM1 is the key event after combinational treatment to induce G2/M arrest. As a key oncogene for multiple cancers, FOXM1 inhibitors have been developed to treat cancers^40^. Our findings provide a new drug combination to inhibit FOXM1 in CRC.

In summary, our preclinical data suggest that blockade of the NF-κB pathway with Bortezomib could render CRC more sensitive to BET inhibition in vitro and in vivo, through repression of FOXM1 to induce G2/M arrest, and repression of c-myc expression to subsequently increase GADD45 proteins and anti-angiogenic factors CTGF and Tsp-1 (Fig. 6f). These findings provide a rational basis for the clinical use of this combination for the treatment of CRC patients, even for those who is resistant to conventional FOLFOX therapy.

## Materials and methods

### Cell culture

All human CRC cell lines were obtained from the American Type Culture Collection (ATCC). Cells were cultured in Dulbecco’s modified Eagle’s medium (DMEM; Hyclone, Logan, UT, USA) supplemented with 10% fetal bovine serum (Hyclone, Logan, UT, USA) and 1% penicillin/streptomycin, and maintained at 37 °C in incubator under an atmosphere containing 5% CO_2_.

### Cell viability assay

CCK8 method was used to investigate the cell viability assay. Cells were seeded in 96-well plates (10^3^ cells, 100 µl per well). The next day, cells were exposed to various agents dissolved in another 100 μl culture medium. Seventy-two hours later, each well was added with 10 μl CCK8 reagent (Dojindo, Washington, USA) and incubated at 37 °C for another 2 h. We measured the absorbance at 450 nm with a spectrophotometer. Synergistic effect of combined treatment was evaluated by isobolographic method of Chou and Talalay^11^ using the Calcusyn software program. CI > 1 indicates additive effect, and CI < 1 indicates synergistic effect. The chemicals used in this study have been included in the Supplementary Table 1.

### Western blotting, qPCR, and siRNA transfection

Western blotting, qPCR, and siRNA transfection were performed as previously described^41^. The antibodies and primers used in this study have been included in the Supplementary Table 1.

### Apoptosis assay

Apoptosis was measured by Pharmingen^TM^ Annexin V Apoptosis Detection Kit (BD Biosciences, Rockville, USA) according to the manufacturer’s instructions, and analyzed by flow cytometry after incubation for 20 min.

### Cell cycle assay

Cells were seeded in 6-well plates and allowed to attach overnight. After treated with indicated agents for 48 h, cells were fixed with 75% ethanol, stained with PI contained with RNase (BD Biosciences, Rockville, USA), and analyzed by flow cytometry after incubation for 20 min.

### RNA-seq analysis

After CRC cells were treated with DMSO, 1 µM JQ1, 5 nM Bortezomib or combination for 6 h, total RNA was extracted. RNA quality was checked using Agilent Bioanalyzer. RNA-seq were performed by HiSeq 2500. Differnetial expressed genes were analyzed by the Deseq2 software. *P* < 0.05 was considered as statistically significant. The data have been deposited in GEO (GSE95513).

### Lentiviral shRNA constructs

shRNA sequences targeting CTGF, GADD45B, and GADD45G were cloned in a pLKO.1 vector. Lentiviruses were generated in 293T cells by using pMD.2G and psPAX2 packaging system. Cells were incubated with supernatant with virus for 2 days and stable pool cells were selected for 1 week by using puromycin.

### NF-kB activity assays

Cells were co-transfected with NF-kB reporter plasmid (Beyotime, China) and Renilla plasmid. After 48 h, media was replaced with fresh media containing indicated reagents. After incubation for another 24 h, luciferase activities were detected by using the Dual-Glo Luciferase Reporter kit according to the manufacturer’s instructions (Promega, Madison, USA).

### CRC cell line xenograft assay

All animal procedures were approved by the animal care and use committees of Xinhua Hospital. Female nude mice (4–6 weeks old) were injected subcutaneously with CRC cell lines (2 × 10^6^). When the tumors became palpable (30–50 mm^3^), mice were randomly divided into four groups. The groups were treated with vehicle (control), JQ1 (50 mg/kg, intraperitoneal administration, q.d.), Bortezomib (1 mg/kg, intraperitoneal administration, b.i.w.), or combination. Tumors were measured two to three times a week. Tumor volumes were calculated as follows: 0.5 × length × width^2^. Animals were monitored for significant adverse effects. Tumors were removed from killed mice, photographed, and paraffin preserved after 18–27 days of treatment. Tumor inhibition rate = (control group volume − treatment group volume)/control group volume × 100%. Tumor inhibition rate >70% was considered as complete responsive treatment (++), according to response evaluation criteria in solid tumors (RICIST) criterion^36^. Tumor inhibition rate <30% was considered as non-responsive treatment (−). Tumor inhibition rate at 30–70% was considered as partial responsive treatment (+).

### Generation of PDXs from colorectal tumors

Human samples for PDX model were collected in the Department of Colorectal Surgery, XinHua Hospital during 2016. Institutional review board approval and informed consent were obtained for all collections.

Tumor tissues, which derived from freshly resected colon or rectum, were intensively washed and cut into 2- to 3-mm^3^ pieces in antibiotic-containing PBS medium. When nude mice (4–6 weeks old, female) were anesthetized with pentobarbital and cut by a small incision in one side of axilla, tumor pieces were implanted subcutaneously. After incubation for 8–16 weeks, tumors were harvested when they reached a volume of 1500 mm^3^ (P1 xenografts). Then tumors from P1 xenografts were cut into small pieces again and implanted subcutaneously to obtain P2 xenografts as previously described. This process was further repeated and the animal studies were performed on P3 or P4 xenografts.

### Immunohistochemistry

Immunohistochemistry analysis was performed as previously described^41^. Expression index = % of positive cells × staining intensity (1+2+ or 3+).

### Statistical analysis

Statistical analysis was performed using SPSS 13.0 software. Results were analyzed by using a two-tailed Students’ *t* test for two groups’ comparison. *P* value <0.05 was considered as statistically significant. The data shown in this study represent the mean ± SD (*p < 0.05, **p < 0.01).

## Electronic supplementary material

Supplementary Figures and Figure legend

Supplementary table 1

## References

[1] Vogelstein B (2013). Cancer genome landscapes. Science.

[2] Brancato JM (2016). BET protein inhibition blocks growth of triple-negative breast cancer by inducing mitotic and cytokinetic dysfunction. Cancer Res..

[3] Delmore JE (2011). BET bromodomain inhibition as a therapeutic strategy to target c-Myc. Cell.

[4] Dawson MA (2011). Inhibition of BET recruitment to chromatin as an effective treatment for MLL-fusion leukaemia. Nature.

[5] Munshi NC, Anderson KC (2013). New strategies in the treatment of multiple myeloma. Clin. Cancer Res..

[6] Barone G, Anderson J, Pearson AD, Petrie K, Chesler L (2013). New strategies in neuroblastoma: therapeutic targeting of MYCN and ALK. Clin. Cancer Res..

[7] Shu S (2016). Response and resistance to BET bromodomain inhibitors in triple-negative breast cancer. Nature.

[8] Mazur PK (2015). Combined inhibition of BET family proteins and histone deacetylases as a potential epigenetics-based therapy for pancreatic ductal adenocarcinoma. Nat. Med..

[9] Mccleland, M. L. et al. CCAT1 is an enhancer-templated RNA that predicts BET sensitivity in colorectal cancer. *J. Clin. Invest.* **126**, 639–652 (2016). 10.1172/JCI83265 PMC4731162 26752646

[10] Kane, R. C, Bross, P. F, Farrell, A. T. & Pazdur, R. Velcade®: US FDA approval for the treatment of multiple myeloma progressing on prior therapy. *Oncologist* **8**, 508–513 (2003). 10.1634/theoncologist.8-6-508 14657528

[11] Chou TC (2010). Drug combination studies and their synergy quantification using the chou-talalay method. Cancer Res..

[12] Tsuchida Y, Therasse P (2001). Response evaluation criteria in solid tumors (RECIST): new guidelines. Pediatr. Blood Cancer.

[13] Costa RH (2005). FoxM1 dances with mitosis. Nat. Cell. Biol..

[14] Rishi, A. K. et al. Post-transcriptional regulation of the DNA damage-inducible gadd45 gene in human breast carcinoma cells exposed to a novel retinoid CD437. *Nucleic Acids Res.* **27**, 3111–3119 (1999). 10.1093/nar/27.15.3111 PMC148537 10454607

[15] Zerbini LF (2004). NF-κB-mediated repression of growth arrest- and DNA-damage-inducible proteins 45α and γ is essential for cancer cell survival. Proc. Natl Acad. Sci. USA.

[16] Marhin WW, Chen S, Facchini LM, Fornace AJ, Penn LZ (1997). Myc represses the growth arrest gene gadd45. Oncogene.

[17] Barsytelovejoy D, Mao DYL, Penn LZ (2004). c-Myc represses the proximal promoters of GADD45a and GADD153 by a post-RNA polymerase II recruitment mechanism. Oncogene.

[18] Caravita T, de Fabritiis P, Palumbo A, Amadori S, Boccadoro M (2006). Bortezomib: efficacy comparisons in solid tumors and hematologic malignancies. Nat. Clin. Pract. Oncol..

[19] Kim DW (2000). The RelA NF-kappaB subunit and the aryl hydrocarbon receptor (AhR) cooperate to transactivate the c-myc promoter in mammary cells. Oncogene.

[20] Huang B, Yang XD, Zhou MM, Ozato K, Chen LF (2009). Brd4 coactivates transcriptional activation of NF-kappaB via specific binding to acetylated RelA. Mol. Cell. Biol..

[21] Zou Z (2013). Brd4 maintains constitutively active NF-κB in cancer cells by binding to acetylated RelA. Oncogene.

[22] Arora R (2014). Panepoxydone targets NF-kB and FOXM1 to inhibit proliferation, induce apoptosis and reverse epithelial to mesenchymal transition in breast cancer. PLoS ONE.

[23] Zhang Z (2016). BET bromodomain inhibition as a therapeutic strategy in ovarian cancer by downregulating FoxM1. Theranostics.

[24] Dews M (2006). Augmentation of tumor angiogenesis by a Myc-activated microRNA cluster. Nat. Genet..

[25] Dang, C. V. MYC on the path to cancer. *Cell* **149**, 22–35 (2012). 10.1016/j.cell.2012.03.003 PMC3345192 22464321

[26] Erisman MD (1985). Deregulation of c-myc gene expression in human colon carcinoma is not accompanied by amplification or rearrangement of the gene. Mol. Cell. Biol..

[27] Sikora K (1987). c-myc oncogene expression in colorectal cancer. Cancer.

[28] Network CGA (2012). Comprehensive molecular characterization of human colon and rectal cancer. Nature.

[29] Sansom OJ (2007). Myc deletion rescues Apc deficiency in the small intestine. Nature.

[30] Togel L (2016). Dual targeting of bromodomain and extraterminal domain proteins, and WNT or MAPK signaling, inhibits c-MYC expression and proliferation of colorectal cancer cells. Mol. Cancer Ther..

[31] Loven, J. et al. Selective inhibition of tumor oncogenes by disruption of super-enhancers. *Cell* **153**, 320–334 (2013).. 10.1016/j.cell.2013.03.036 PMC3760967 23582323

[32] Cloney, R. Gene expression: dynamic enhancer-promoter interactions for transcriptional bursting. *Nat. Rev. Genet.* **17**, 437 (2016). 10.1038/nrg.2016.81 27320128

[33] Zhan Q, Bae I, Kastan MB, Fornace AJ (1994). The p53-dependent gamma-ray response of GADD45. Cancer Res..

[34] Zerbini LF (2004). NF-κB-mediated repression of growth arrest-and DNA-damage-inducible proteins 45α and γ is essential for cancer cell survival. Proc. Natl Acad. Sci. USA.

[35] Baudino TA (2002). c-Myc is essential for vasculogenesis and angiogenesis during development and tumor progression. Genes Dev..

[36] Yoshiaki Tsuchida MD, Patrick Therasse MD (2001). Response evaluation criteria in solid tumors (RECIST): New guidelines. Med. Pediatr. Oncol..

[37] Mackay H (2005). A phase II trial with pharmacodynamic endpoints of the proteasome inhibitor bortezomib in patients with metastatic colorectal cancer. Clin. Cancer Res..

[38] Felsher DW, Zetterberg A, Zhu J, Tlsty T, Bishop JM (2000). Overexpression of MYC causes p53-dependent G2 arrest of normal fibroblasts. Proc. Natl Acad. Sci. USA.

[39] Wang XW (1999). GADD45 induction of a G2/M cell cycle checkpoint. Proc. Natl Acad. Sci. USA.

[40] Gormally MV (2014). Suppression of the FOXM1 transcriptional programme via novel small molecule inhibition. Nat. Commun..

[41] Liu Y (2016). Increased TEAD4 expression and nuclear localization in colorectal cancer promote epithelial-mesenchymal transition and metastasis in a YAP-independent manner. Oncogene.

