# Supplementary material for: Co-inhibition of BET proteins and NF-κB as a potential therapy for colorectal cancer through synergistic inhibiting MYC and FOXM1 expressions
Source: Cell Death Dis. 2018 Feb 22;9(3):315. doi: 10.1038/s41419-018-0354-y (PMC5833769; doi:10.1038/s41419-018-0354-y)
Supplement: Supplementary file 1 — Supplementary Figures and Figure legend [file 41419_2018_354_MOESM1_ESM.pdf]

Supplementary Fig.1

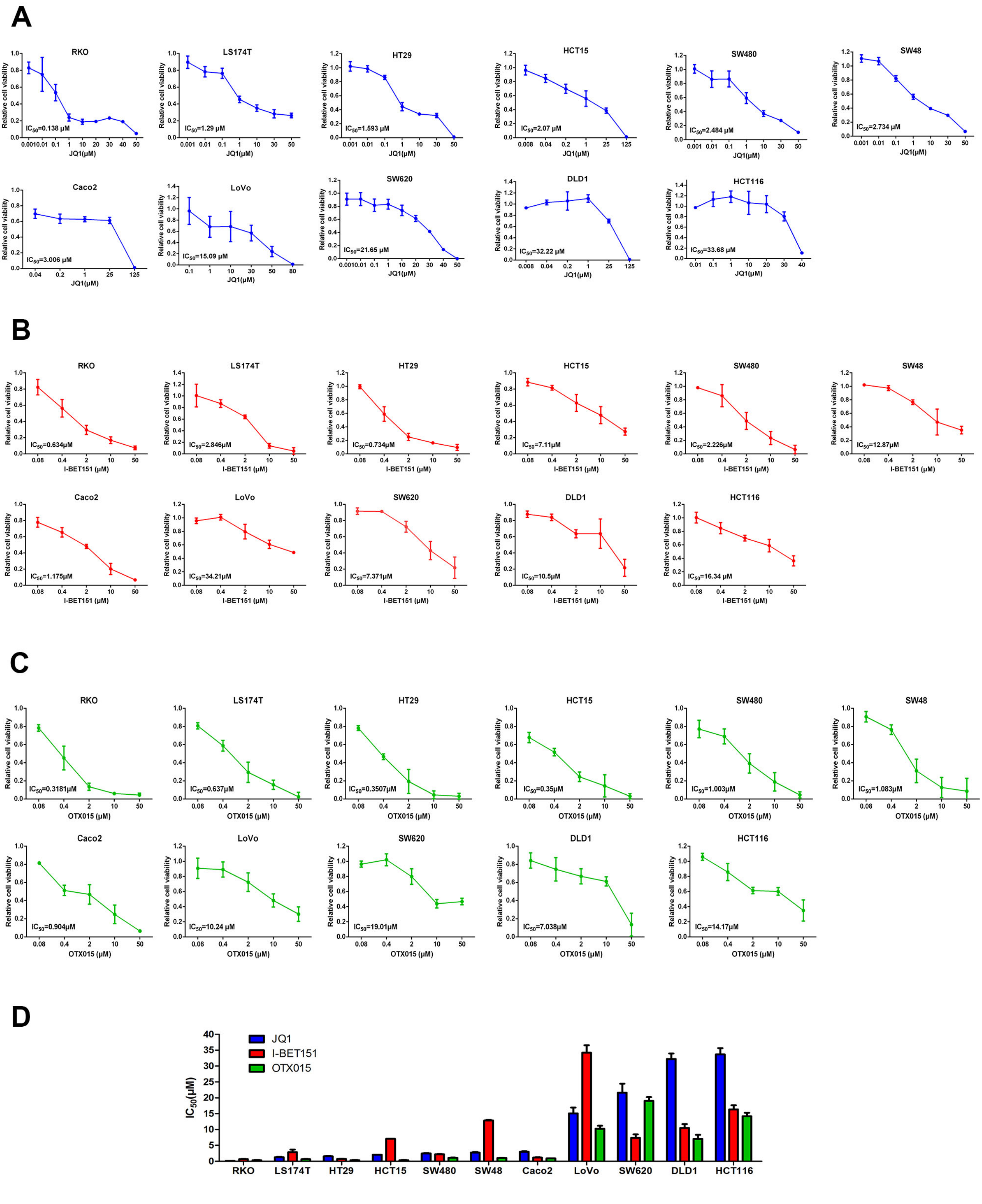

Supplementary Fig.2

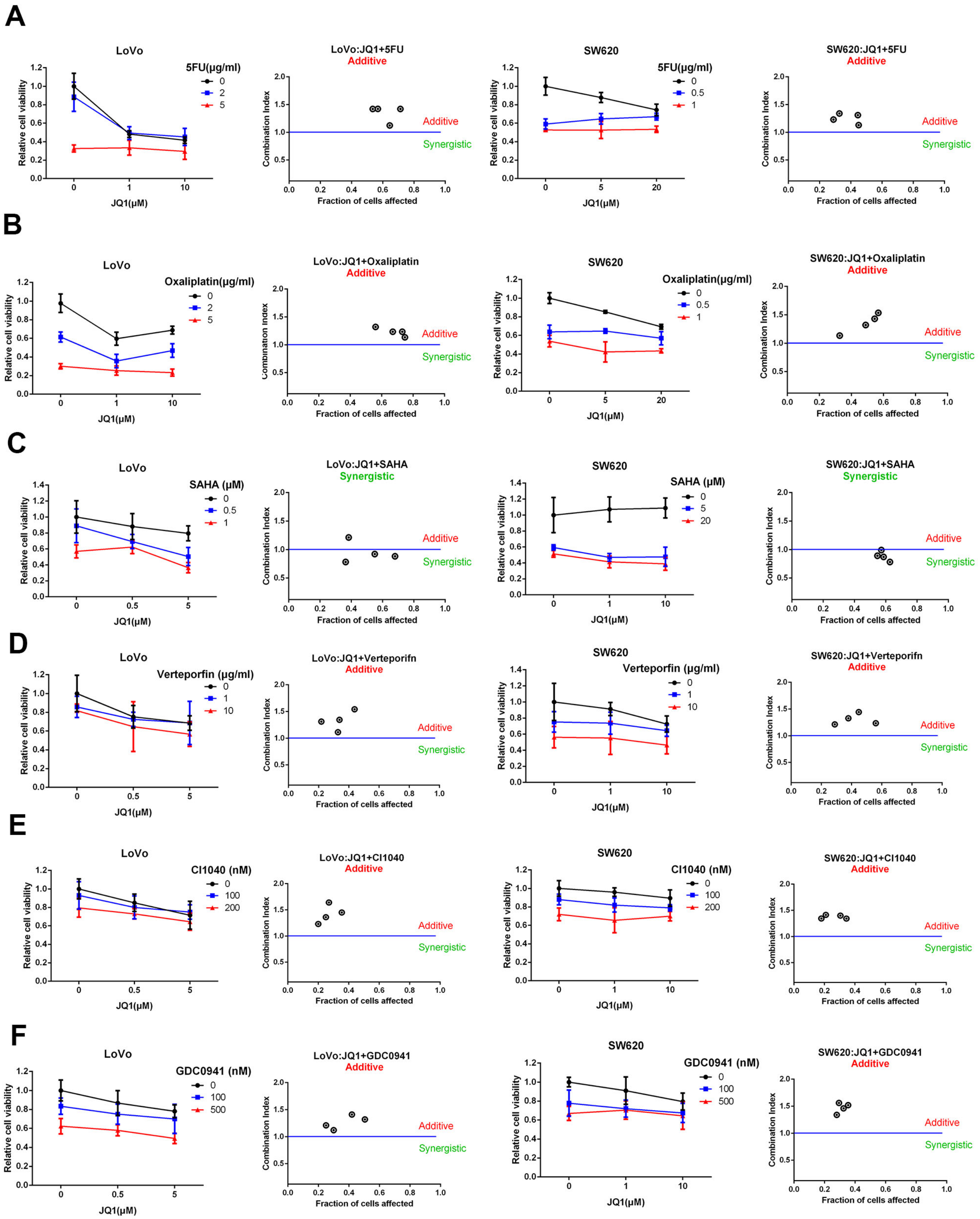

Supplementary Fig.3

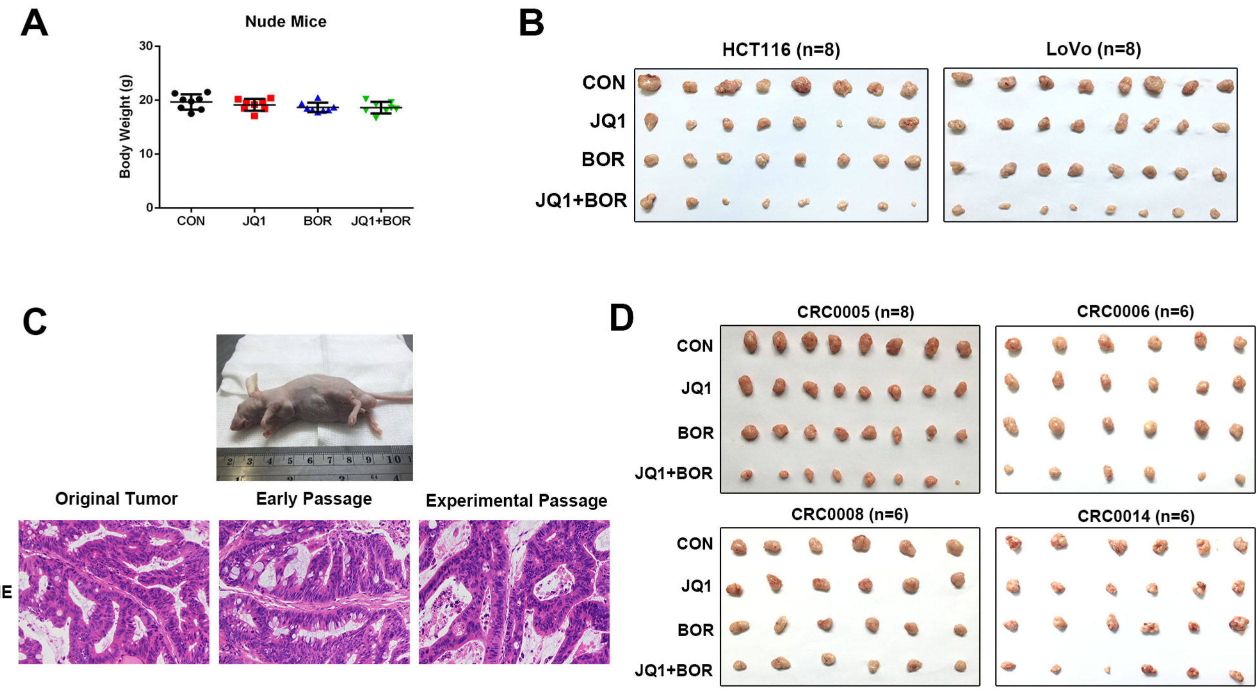

E

Number	Gender	Age	Tumor	Type	TNM Staging	Differentiation	FOLFOX6 therapy	JQ1	BOR	JQ1+ BOR
CRC0005	female	61	rectum	adenocarcinoma	Ⅱ	Ⅱ	sensitive	+	+	++
CRC0006	female	82	colon	mucinous adenocarcinoma	Ⅲ	Ⅲ	sensitive	+	-	++
CRC0008	male	73	rectum	adenocarcinoma	Ⅲ	Ⅱ	resistant	-	-	+
CRC0014	male	71	rectum	adenocarcinoma	Ⅲ	Ⅲ	resistant	-	-	++

F

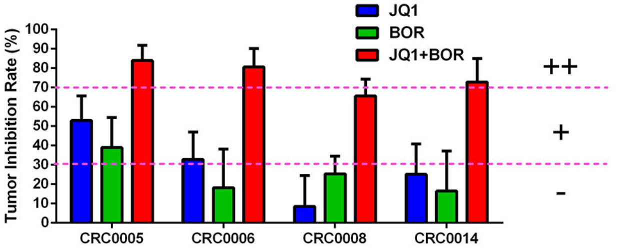

Supplementary Fig.4

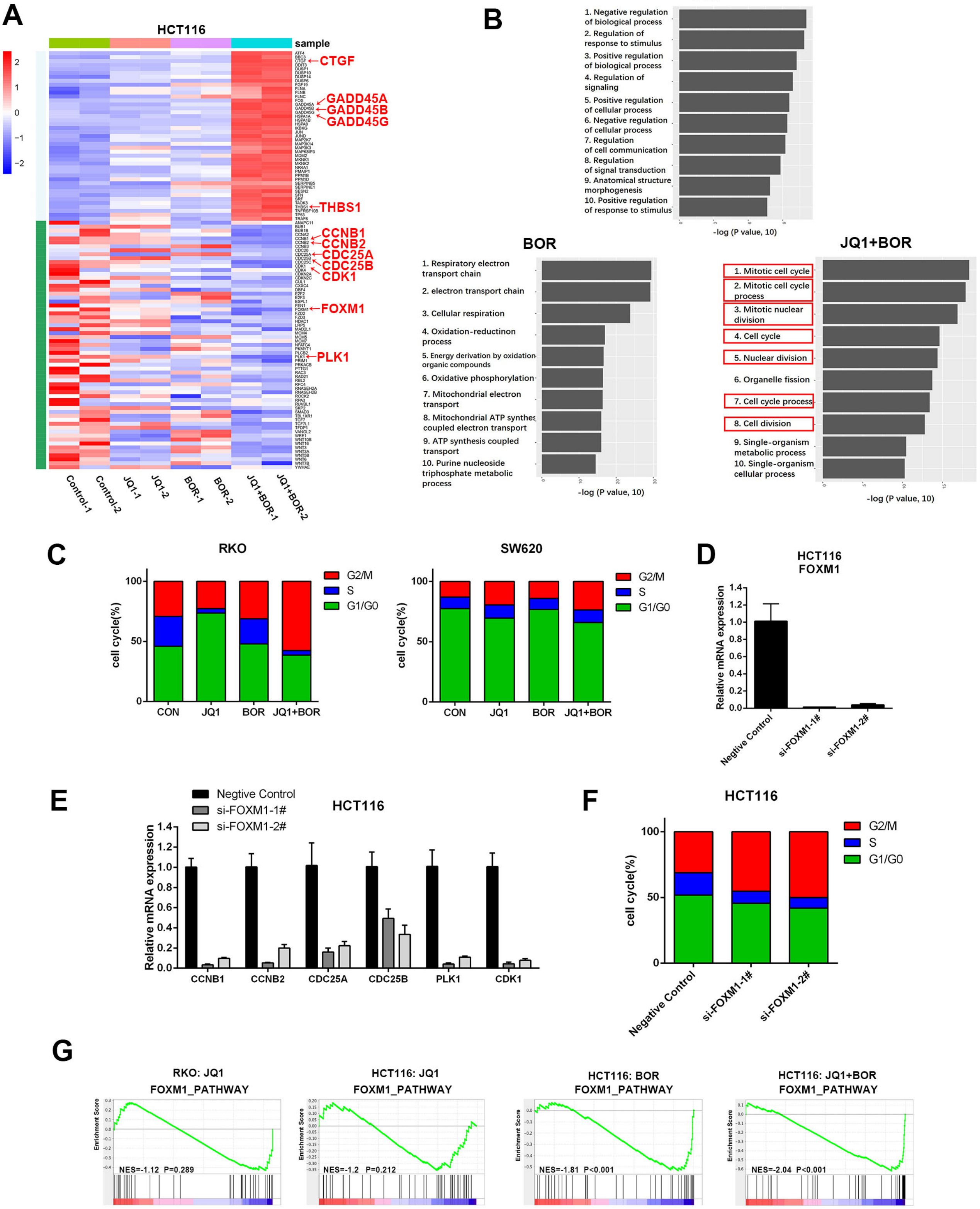

Supplementary Fig.5

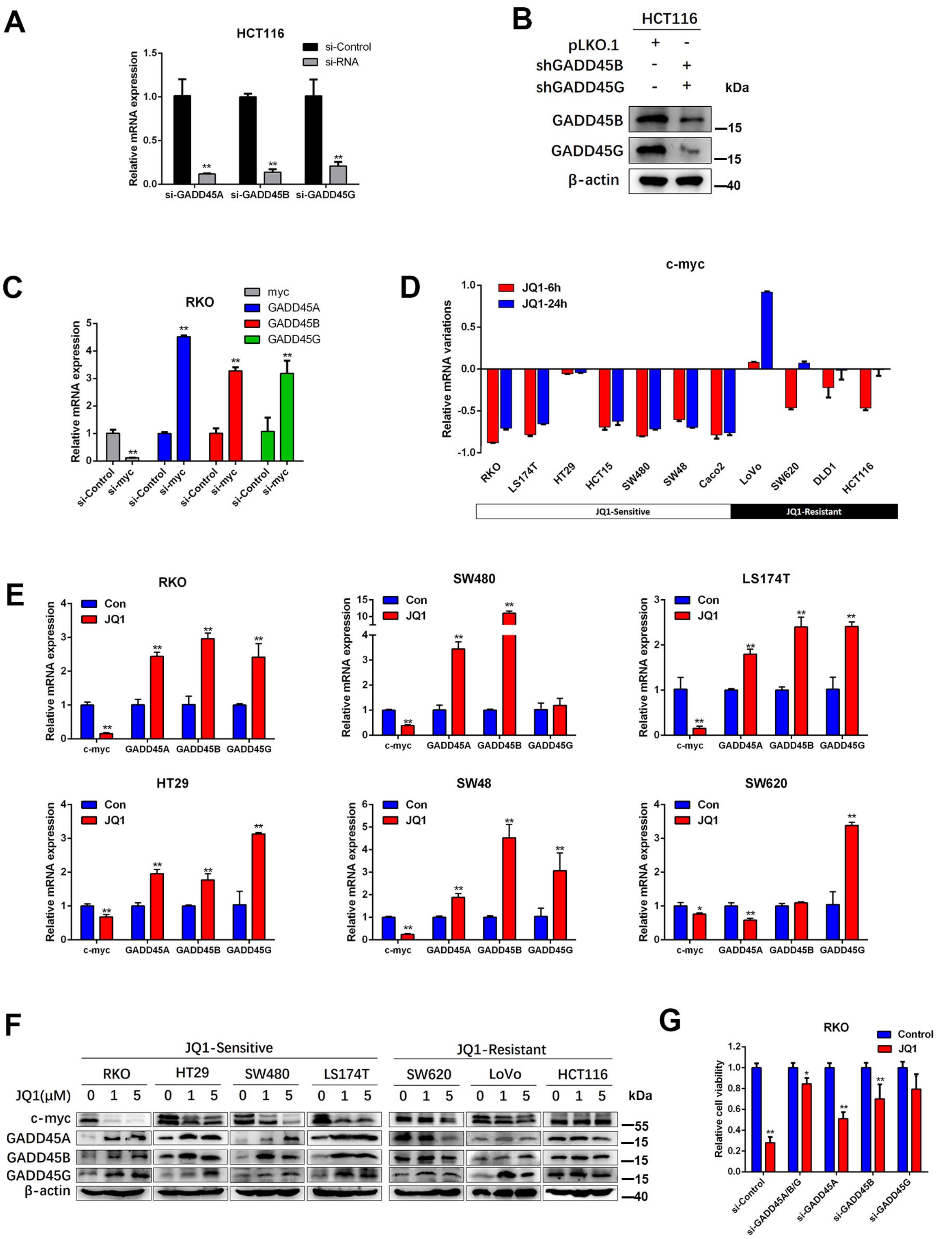

Supplementary Fig.6

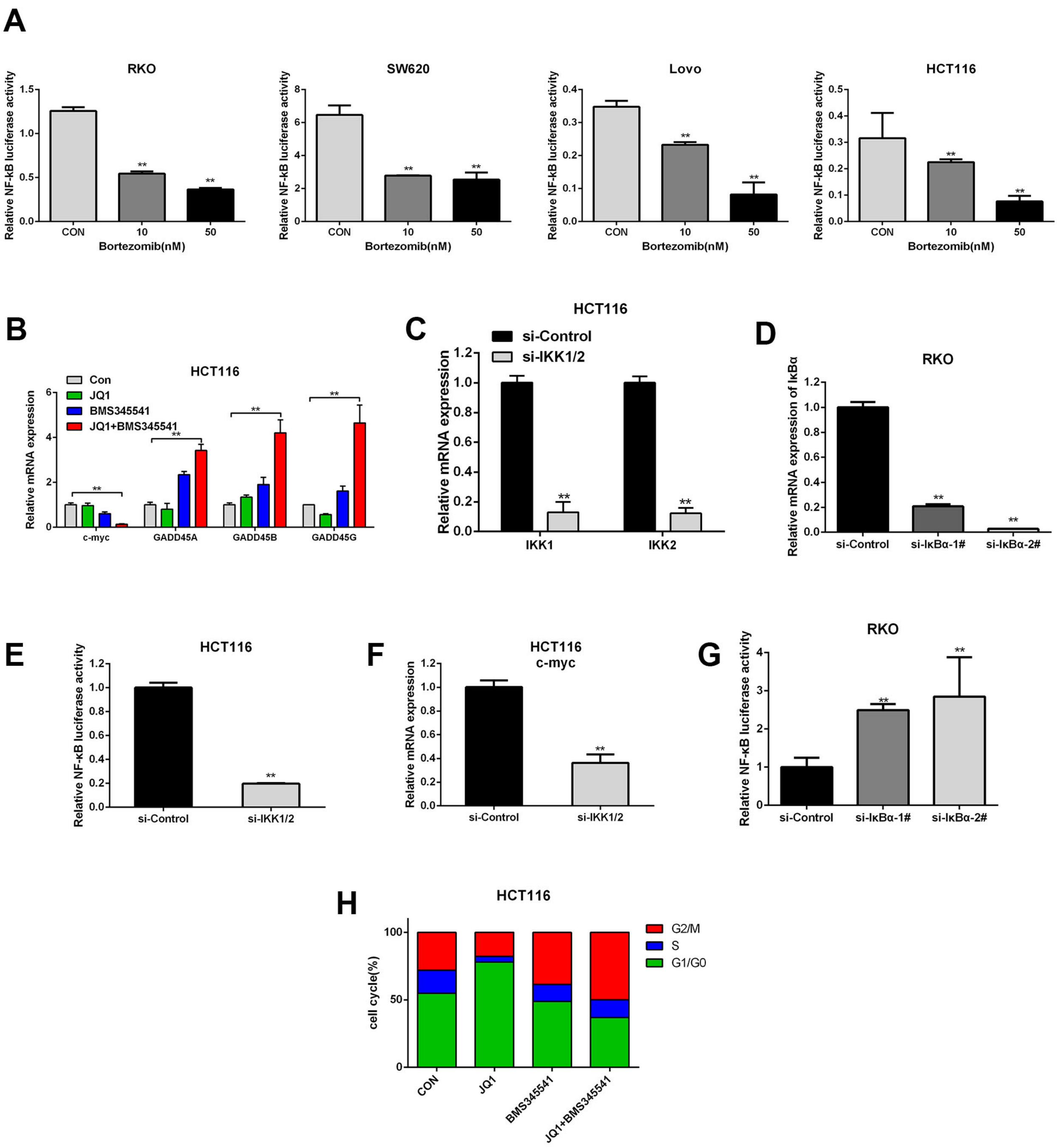

Supplementary Fig.7

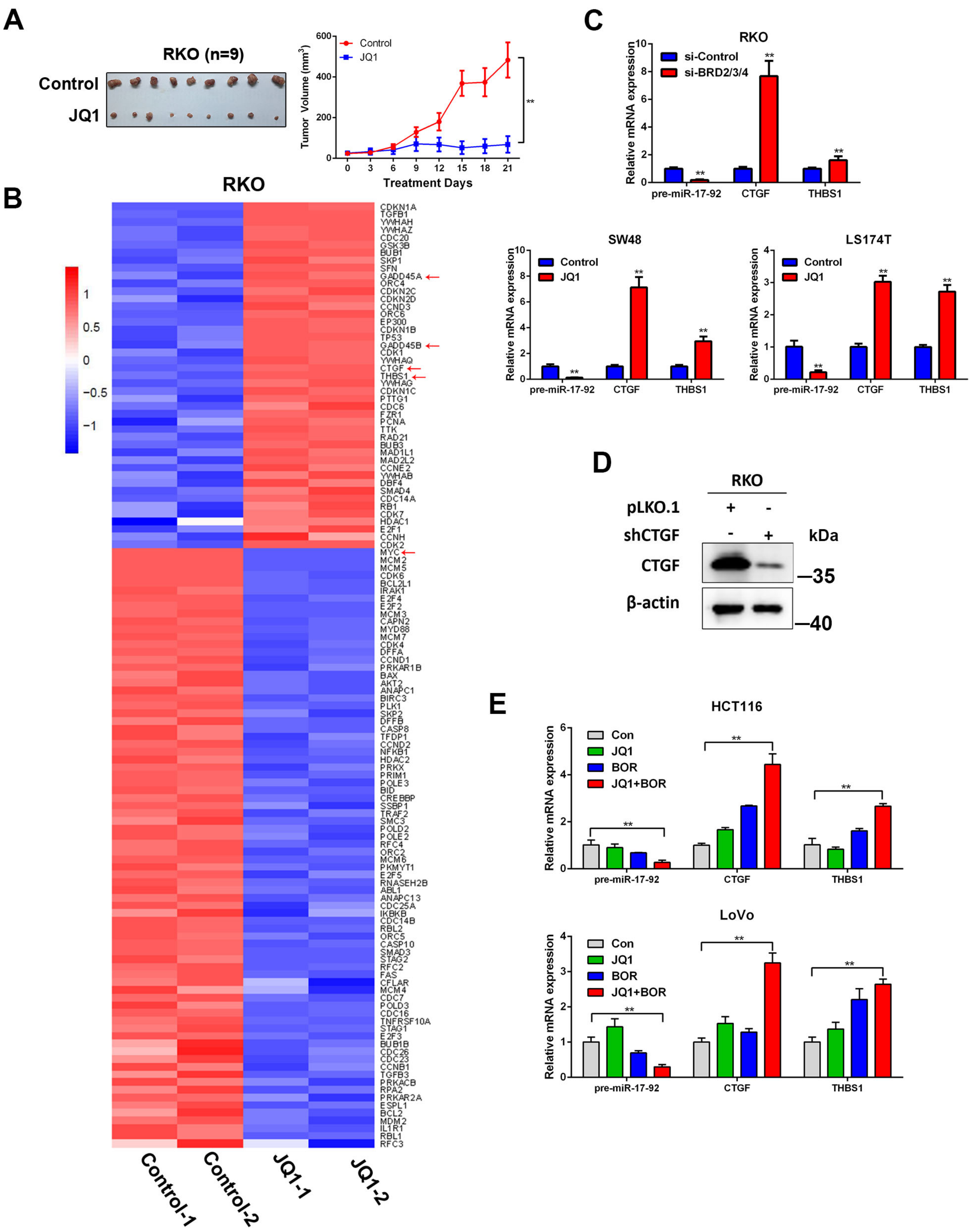

Supplementary Fig.8

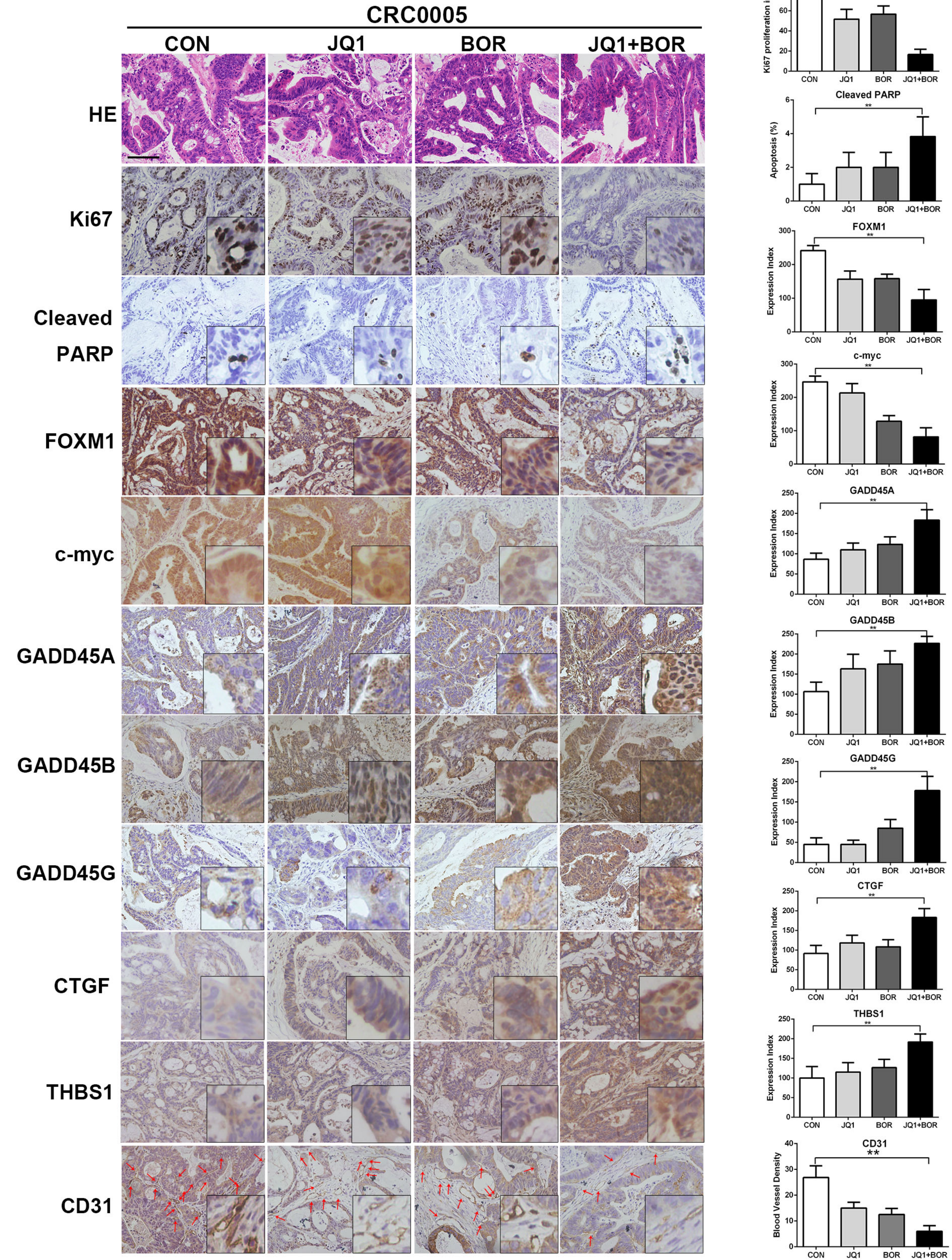

Supplementary Figure 1. Cell viability of CRC cells treated with BETi. CRC cells were treated with JQ1 (A), I-BET151 (B) and OTX015 (C) for 72h. Cell viability was measured by CCK8. (D) IC50 values of 11 CRC cells treated with JQ1, I-BET151 and OTX015.

Supplementary Figure 2. Drug screening for effective combination therapies in BETi-resistant cells. (A-F) BETi-resistant cells were treated concurrently with JQ1 and 5FU, oxaliplatin, SAHA, verteporfin, CI1040, GDC0941 at the indicated concentrations for 72h. Cell viability was measured by CCK8. The synergistic cytotoxicity was quantitatively analyzed by Combination Index (CI) using the Calcusyn software program. Each dot represented one combinational treatment group. The data shown represent the mean ± SD.

Supplementary Figure 3. JQ1 and Bortezomib co-treatment inhibit tumor growth of CRC cell lines- and patients-derived xenografts (PDX). (A) Xenograft tumors of HCT116 and LoVo cells treated with JQ1 and Bortezomib for 18 days. Body weights of nude mice were measured on the last day of treatment. All mice showed tolerance to treatment and maintained normal activities, with no marked changes in body weight by the end of drug treatment. (B) Representative images of Xenograft tumors of HCT116 and LoVo cells treated with JQ1, Bortezomib and JQ1+Bortezomib on the last day of treatment (n=8 per group). (C) Representative H&E staining images of the original tumors and

of xenografts at the early passage (Px1) and the passage used for the experiments (Px3). (D) Representative images of Xenograft tumors of CRC PDXs treated with JQ1, Bortezomib and JQ1+Bortezomib on the last day of treatment (E) Clinical pathological features of the PDXs. (F) The tumor inhibition rate of 4 PDXs treated with JQ1, Bortezomib and JQ1+Bortezomib.

Supplementary Figure 4. JQ1 and Bortezomib treatment induce G2/M arrest through inhibition FOXM1 expression in CRC cells. (A) Heatmap of significantly differentially expressed genes in HCT116 cells treated with JQ1 (1μM), Bortezomib (5nM) and JQ1+Bortezomib for 6 h. (B) Gene ontology analysis of GSEA analysis of gene expression profile of HCT116 cells treated with JQ1+Bortezomib compared to the vehicle control. (C) RKO and SW620 cells were treated with indicated compounds for 24h. The cell cycle was analyzed by PI staining. (D) HCT116 cells were transfected with FOXM1 siRNA for 3 days. The knockdown efficiency of FOXM1 was analyzed by qPCR. (E) The gene expression of G2/M checkpoint genes after FOXM1 knockdown were analyzed by qPCR. (F) HCT116 cells were transfected with FOXM1 siRNA for 3 days. The cell cycle was analyzed by PI staining. (G) GSEA analysis of FOXM1 pathway enrichment in the HCT116 cells treated with indicated compounds.

Supplementray Figure 5. GADD45 plays a critical role in synergistic cytotoxic effect. (A) The knockdown efficiency of GADD45A/B/G was confirmed by qPCR.

(B) Western blot analysis of pLKO.1 vector and shGADD45B/G HCT116 cells. (C) mRNA alteration of GADD45A/B/G in RKO cells transfected with control siRNA and c-myc siRNA for 3 days. (D) Relative mRNA variation of c-myc in the 11 CRC cells treated with JQ1 (1μM). (E, F) mRNA and protein expression of c-myc, GADD45A/B/G in a panel of CRC cells treated with JQ1 (1μM) for 24h. (G) RKO cells were transfected with GADD45A/B/G siRNA for 2 days, then cells were treated with JQ1 (1μM) for another 72h. Cell viability was measured by CCK8.

Supplementary Figure 6. (A) Bortezomib treatment inhibits NF-κB reporter activity in a dose dependent manner. (B) mRNA alteration of c-myc and GADD45A/B/G in HCT116 cells treated with JQ1(1μM), BMS345541 (2μM) or JQ1+ BMS345541 for 24h. (C) The knockdown efficiency of IKKs was confirmed by qPCR. (D) The knockdown efficiency of IκBα was confirmed by qPCR. (E) NF-κB reporter assay of HCT116 cells transfected with IKK1/2 siRNA. (F) qPCR analysis of c-myc expression in HCT116 cells transfected with IKK1/2 siRNA. (G) NF-κB reporter assay of RKO cells transfected with two individual IκBα siRNA. (H) HCT116 cells were treated with JQ1(1μM), BMS345541 (2μM) or JQ1+ BMS345541 for 24h. The cell cycle was analyzed by PI staining.

Supplementary Figure 7. JQ1 treatment leads to upregulation of CTGF and THBS1 in JQ1-sensitve cells. (A) Xenograft growth curves of RKO cells treated with JQ1 (50 mg/kg, daily) or vehicle control for 21 days. (B) Heatmap of

significantly differentially expressed genes in RKO cells treated with JQ1 (1 μM) for 6 h. (C) mRNA alteration of pre-miR-17-92, CTGF and THBS1 in JQ1-sensitive cells treated with JQ1 (1 μM) for 24h or transfected with BRD2/3/4 siRNA for 3 days. (D) Western Blot validation of CTGF knockdown in the shCTGF RKO stable cells. (E) mRNA alteration of pre-miR-17-92, CTGF and THBS1 in JQ1-resistant cells treated with JQ1(1μM), Bortezomib(5nM) or JQ1+Bortezomib for 24h. The data shown represent the mean ± SD. ($p<0.05$, *; $p<0.01$, **).

Supplementary Figure 8. JQ1 and Bortezomib co-treatment show synergistically growth inhibition in the CRC patients-derived xenografts (PDX). Immunohistochemical staining of Ki67, cleaved PARP, c-myc, GADD45A/B/G, FOXM1, CTGF, THBS1, CD31 in the PDX CRC0005. Bars, 100μm. The data shown represent the mean ± SD. ($p<0.05$, *; $p<0.01$, **).
