# Supplementary material for: Co-inhibition of BET proteins and NF-κB as a potential therapy for colorectal cancer through synergistic inhibiting MYC and FOXM1 expressions
Source: Cell Death Dis. 2018 Feb 22;9(3):315. doi: 10.1038/s41419-018-0354-y (PMC5833769; doi:10.1038/s41419-018-0354-y)
Supplement: Supplementary file 2 — Supplementary table 1 [file 41419_2018_354_MOESM2_ESM.doc]

| **Gene** | **Sense** | **Antisense** |
| --- | --- | --- |
| c-myc | CCTTCTCTCCGTCCTCGGAT | CTTGTTCCTCCTCAGAGTCGC |
| GADD45A | AGCTCCTGCTCTTGGAGACC | GCAGGATCCTTCCATTGAGA |
| GADD45B | ACAGTGGGGGTGTACGAGTC | GATGTCATCCTCCTCCTCCTC |
| GADD45G | ACTAGCTGCTGGTTGATCGC | CAACTCATGCAGCGCTTTC |
| Pre-miR-17-92 | CTGTCGCCCAATCAAACTG | GTCACAATCCCCACCAAAC |
| IKK1 | TTTGAAGCAGCCAAGATGTTT | GAATGAAGACTTTCATCAGGTGG |
| IKK2 | AAATGAAAGAGCGCCTTGG | CACTGCTTGATGGCAATCTG |
| β-Actin | GCACAGAGCCTCGCCTT | GTTGTCGACGACGAGCG |
| IκBα | GTCAAGGAGCTGCAGGAGAT | TCATGGATGATGGCCAAGT |
| THBS1 | CAATGCCACAGTTCCTGATG | CACAGCTCGTAGAACAGGAGG |
| CTGF | CAGGCTAGAGAAGCAGAGCC | TGGAGATTTTGGGAGTACGG |
| BRD2 | CTACGTAAGAAACCCCGGAAG | GCTTTTTCTCCAAAGCCAGTT |
| BRD3 | CCTCAGGGAGATGCTATCCA | ATGTCGTGGTAGTCGTGCAG |
| BRD4 | AGCAGCAACAGCAATGTGAG | GCTTGCACTTGTCCTCTTCC |

**Primers used in this paper**

**siRNA used in this paper**

| **Gene** | **Sense** |
| --- | --- |
| GADD45A | AACGTCGACCCCGATAACGTG |
| GADD45B | AAGTTGATGAATGTGGACCCA |
| GADD45G | AACGAGGACGCCTGGAAGGAT |
| IKK1 | GCAGGCUCUUUCAGGGACA |
| IKK2 | GGUGAAGAGGUGGUGGUGAGC |
| IκBα-1# | GCCAGAAAUUGCUGAGGCA |
| IκBα-2# | GAGUCAGAGUUCACGGAGU |
| BRD2 | GCCGAGUUGUGCAUAUAAUTT |
| BRD3 | GGGAGAUGCUAUCCAAGAATT |
| BRD4 | GGAGAUGACAUAGUCUUAATT |
| c-myc | GUGCAGCCGUAUUUCUACUTT |

**shRNA used in this paper**

| **Gene** | **Target sequence** |
| --- | --- |
| GADD45B | AAGTTGATGAATGTGGACCCA |
| GADD45G | AACGAGGACGCCTGGAAGGAT |
| THBS1 | TGACATCAGTGAGACCGATTT |
| CTGF | AAAT CTCCAAGCCTATCAAGT |

**Antibodies used in this paper**

| **Antibodies** | **Corporation** | **Purpose** | **Concentration** |
| --- | --- | --- | --- |
| anti-cleaved PARP1 | Abcam (Cambridge, UK) | IHC | 1:200 |
|  |  | WB | 1:2000 |
| anti-BRD2 | Abcam (Cambridge, UK) | WB | 1:2000 |
|  |  | IF | 1:200 |
| anti-BRD3 | Abcam (Cambridge, UK) | WB | 1:1000 |
|  |  | IF | 1:200 |
| anti-BRD4 | Abcam (Cambridge, UK) | WB | 1:2000 |
|  |  | IF | 1:200 |
| anti-GADD45A | Abcam (Cambridge, UK) | IHC | 1:100 |
|  |  | WB | 1:1000 |
| anti-GADD45B | Abcam (Cambridge, UK) | IHC | 1:50 |
|  |  | WB | 1:1000 |
| anti-GADD45G | Abcam (Cambridge, UK) | IHC | 1:100 |
|  |  | WB | 1:1000 |
| anti-CD31 | Abcam (Cambridge, UK) | IHC | 1:100 |
| anti-αMSA | Abcam (Cambridge, UK) | IHC | 1:100 |
| anti-c-myc | Abcam (Cambridge, UK) | IHC | 1:50 |
|  |  | WB | 1:1000 |
| anti-CTGF | Abclone (Boston, USA) | IHC | 1:50 |
|  |  | WB | 1:2000 |
| anti-THBS1 | Abclone (Boston, USA) | IHC | 1:50 |
|  |  | WB | 1:2000 |
| anti-β-actin | Sigma Aldrich (St. Louis, USA) | WB | 1:20000 |
| anti-ki67 | Dako (Copenhagen, DM) | IHC | 1:1000 |
| anti-β-tubulin | CST (Beverly, USA) | WB | 1:5000 |

**Cytotoxic agents used in this paper**

| **Cytotoxic agents** | **Corporation** |
| --- | --- |
| JQ1 | Selleck (Houston, USA) |
| Bortezomib | Selleck (Houston, USA) |
| BMS345541 | Selleck (Houston, USA) |
| SAHA | MedChem Express (Newark, USA) |
| GDC0941 | MedChem Express (Newark, USA) |
| 5FU | Sigma Aldrich (St. Louis, USA) |
| Oxaliplatin | Sigma Aldrich (St. Louis, USA) |
| Verteporifn | Sigma Aldrich (St. Louis, USA) |
